# Heterodinuclear Mg(II)M(II) (M=Cr, Mn, Fe, Co, Ni, Cu and Zn) Complexes for the Ring Opening Copolymerization of Carbon Dioxide/Epoxide and Anhydride/Epoxide

**DOI:** 10.1002/chem.202104198

**Published:** 2022-02-17

**Authors:** Natalia V. Reis, Arron C. Deacy, Gloria Rosetto, Christopher B. Durr, Charlotte K. Williams

**Affiliations:** ^1^ Department of Chemistry University of Oxford Chemistry Research Laboratory 12 Mansfield Rd Oxford OX1 3TA UK

**Keywords:** anhydride, carbon dioxide, catalysis, epoxide, heterodinuclear, polymerization, synergy

## Abstract

The catalysed ring opening copolymerizations (ROCOP) of carbon dioxide/epoxide or anhydride/epoxide are controlled polymerizations that access useful polycarbonates and polyesters. Here, a systematic investigation of a series of heterodinuclear Mg(II)M(II) complexes reveals which metal combinations are most effective. The complexes combine different first row transition metals (M(II)) from Cr(II) to Zn(II), with Mg(II); all complexes are coordinated by the same macrocyclic ancillary ligand and by two acetate co‐ligands. The complex syntheses and characterization data, as well as the polymerization data, for both carbon dioxide/cyclohexene oxide (CHO) and endo‐norbornene anhydride (NA)/cyclohexene oxide, are reported. The fastest catalyst for both polymerizations is Mg(II)Co(II) which shows propagation rate constants (*k*_p_) of 34.7 mM^−1^ s^−1^ (CO_2_) and 75.3 mM^−1^ s^−1^ (NA) (100 °C). The Mg(II)Fe(II) catalyst also shows excellent performances with equivalent rates for CO_2_/CHO ROCOP (*k*_p_=34.7 mM^−1^ s^−1^) and may be preferable in terms of metallic abundance, low cost and low toxicity. Polymerization kinetics analyses reveal that the two lead catalysts show overall second order rate laws, with zeroth order dependencies in CO_2_ or anhydride concentrations and first order dependencies in both catalyst and epoxide concentrations. Compared to the homodinuclear Mg(II)Mg(II) complex, nearly all the transition metal heterodinuclear complexes show synergic rate enhancements whilst maintaining high selectivity and polymerization control. These findings are relevant to the future design and optimization of copolymerization catalysts and should stimulate broader investigations of synergic heterodinuclear main group/transition metal catalysts.

## Graphical abstract

**A series of new** heterodinuclear complexes comprising Mg(II)M(II) are reported as highly efficient catalysts for both the ROCOP of CO_2_/cyclohexene oxide and norbornene anhydride/cyclohexene oxide. Mg(II)Co(II) and Mg(II)Fe(II) were the most effective combinations in the catalysis.

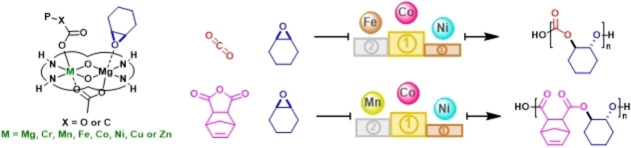

## Introduction

Polyesters and polycarbonates are undergoing a renaissance due to concerns about hydrocarbon polymers’ sustainability and pollution. In contrast to hydrocarbon polymers, which are almost all sourced from petroleum or methane, many of the monomers used to make these oxygenated polymers are, or could be in future, bio‐derived.^[1]^ Polycarbonate production even allows for recycling of waste carbon dioxide and is a front‐runner carbon dioxide utilization technology.^[2]^ These oxygenated polymer chemistries are closer to equilibrium and, as such, may be amenable to complete and selective chain degradation reactions, either to regenerate monomers or, in some cases, to allow for biodegradation.^[3]^

Heteroallene/epoxide Ring Opening Copolymerization (ROCOP) affords an atom economical route to either polyesters (anhydride/epoxide) or polycarbonates (CO_2_/epoxides).^[4]^ With judicious catalyst selection, these processes may show high polymerization control, allowing for predictable degrees of polymerization, high end‐group control and facilitating block polymer preparation.^[5]^ One benefit of these polymerizations is the diverse range of polymer backbones and functionalities accessed from commercial raw materials. For example, ROCOP is an efficient means to make rigid, high glass transition temperature (*T*_g_) polyesters/carbonates, which are challenging to access by alternative heterocyclic ring opening polymerizations.^[6]^ Relevant to this work, the ROCOP of commercially available cyclohexene oxide (CHO) and norbornene anhydride (NA) affords an alternating polyester (PCHNA) showing a *T*_g_ value of 111–116 °C,^[7]^ whilst the *T*_g_ for poly(cyclohexene carbonate) (PCHC from CO_2_/CHO ROCOP) is 105–115 °C.^[8]^ PCHC shows a stress at break (11–42 MPa) and Young's modulus (2400–3600 MPa).^[8b]^ When these polymers are prepared by living polymerization catalysts, it's straightforward to incorporate them into block polymers and to exploit their properties in thermoplastic elastomers, ductile plastics, pressure sensitive adhesives or solution nanostructures for active‐substrate controlled release.^[9]^

**Figure 1:**
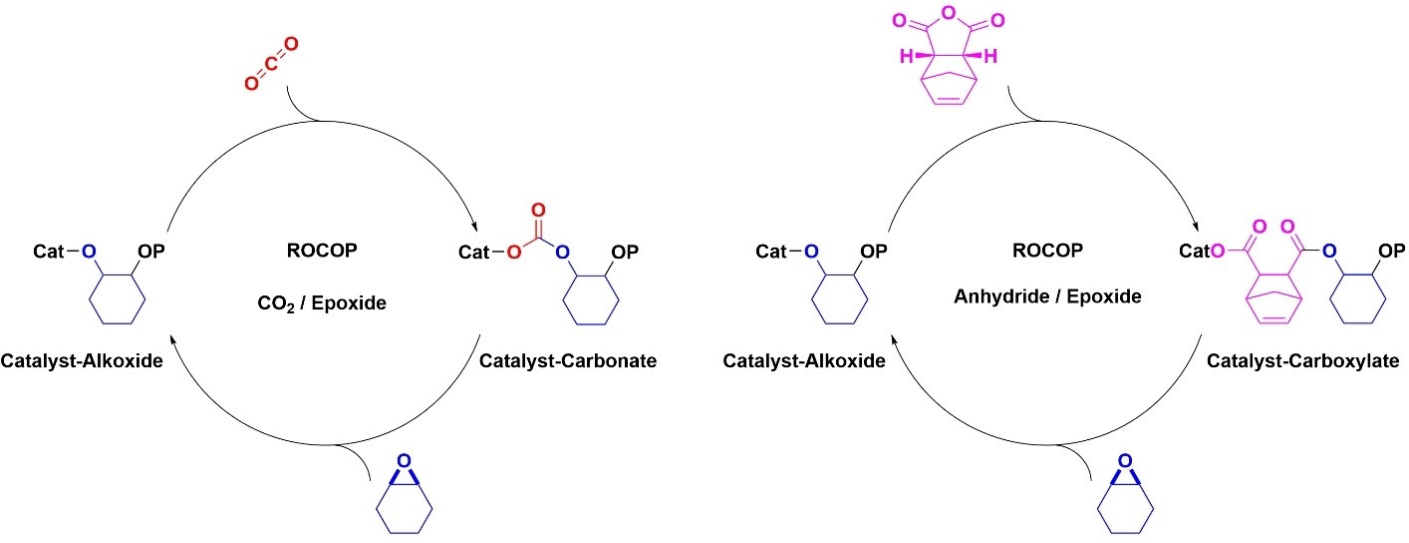
Proposed catalytic cycles, and key intermediates, relevant to the ring opening copolymerization (ROCOP) of CO_2_/cyclohexene oxide and norbornene anhydride/cyclohexene oxide.

Ring opening copolymerization catalytic cycles involve alternation between alkoxide/carbonate or alkoxide/carboxylate intermediates following a series of sequential monomer insertions (Figure 1).[^4b^, ^4c^, ^4d^] Catalysts should be sufficiently Lewis acidic to undergo rapid epoxide coordination, have labile carbonate/carboxylate intermediates and undergo rapid insertions of carbon dioxide/anhydride into the alkoxide intermediate. The best catalysts also show redox stability, apply earth‐abundant elements, are straightforward to prepare, operate at low loadings, show high monomer tolerance and maintain high temperature activity to manage polymer viscosity.[^4b^, ^4c^, ^4d^] Homogeneous catalysts can combine high control, rates and may provide understanding of the elementary polymerization steps. A widely studied class are bicomponent [(salen)MCl]/PPNCl systems, where M=Al(III), Cr(III) or Co(III), and PPNCl is an ionic co‐catalyst essential for high activity and selectivity.^[10]^ These catalysts are easy to synthesize, active for many monomers and simple to apply, but the bicomponent active site (co‐catalyst use) complicates rate laws and limits catalyst loadings; further the PPNCl co‐catalysts tend to be expensive and may be corrosive. The natural evolution of catalyst design to metal salen complexes featuring ligand tethered co‐catalysts dramatically improved performances.^[11]^ For example, a tetra‐substituted catalyst, [(salen[NR_3_^+^]_4_)Co(III)X_5_] (X=DNP=2,4‐dinitrophenolate), showed outstanding activity for CO_2_/propene oxide (PO) (TOF=22 000 h^−1^, 0.001 mol%, 80 °C, 17–20 bar CO_2_) or phthalic anhydride (PA) ROCOP (TOF=1900 h^−1^, [cat]_0_:[PA]_0_:[PO]_0_=1: 7500: 100 000, 80 °C).[^11c^, ^12^] Recently, an amino cyclopropenium (CyPr) substituted Al(III) catalyst, [(salen[CyPr]^+^)AlCl_2_] showed four times higher activity and better stability to chain‐transfer agents than a bicomponent analogue, in NA/propylene oxide ROCOP ([cat]_0_:[NA]_0_:[PO]_0_=1 : 2000 : 10 000, 60 °C).^[13]^ Similarly, an ammonium substituted (NR_4_^+^) porphyrin catalyst [(porphyrin[NR_3_]_4_^+^)Al(III)Cl_5_] for CO_2_/CHO ROCOP, shows a 5‐fold rate enhancement compared with the analogous bicomponent system.[^13a^, ^14^] One drawback of this strategy is the difficulty in synthesizing the complexes, with multi‐step procedures reducing overall complexation yields. Another strategy has been to coordinate two metals by a suitable ancillary ligand to produce dinuclear catalysts; these bimetallic species obviate co‐catalyst requirements. The pioneering work in this field, by Coates and co‐workers, used dinuclear Zn(II)‐*β*‐diiminate catalysts that showed excellent rates in CO_2_/CHO ROCOP.^[15]^ Subsequently, Rieger and co‐workers delivered related di‐Zn(II) catalysts, using deliberately dinucleating β‐diimine ligands, most of which showed activities between 4000 and 10 000 h^−1^ (0.025 mol %, 100 °C, 10–40 bar CO_2_).^[16]^ Multinuclear catalysts also show high activity in either CO_2_/epoxide or anhydride/epoxide ROCOP.^[17]^ Recently, Chen and co‐workers reported a tri‐Co(III) catalyst system [(trisalen)Co_3_(OAc)_3_]/3 PPNCl for CO_2_/PO ROCOP showing high activity (TOF=3000 h^−1^, 0.016 mol%, 60 °C, 30 bar CO_2_) but a very similar tri‐Al(III) complex showed modest activity for phthalic anhydride (PA)/PO ROCOP (TOF=33 h^−1^, 0.03 mol%, 80 °C) when applied without any co‐catalyst.[^17a^, ^17b^] Using the same ligand, Lu and co‐workers reported that [(trisalen)Cr_3_Cl_3_]/3 PPNCl has outstanding activity in PA/CHO ROCOP (TOF=10 620 h^−1^, 0.003 mol%, 100 °C).^[17c]^

Since 2008, our research group has investigated dinuclear heteroallene/epoxide ROCOP catalysts, which operate without co‐catalysts.^[4e]^ Recently, it was discovered that heterodinuclear catalysts, that is, complexes of the form [LM_1_(II)M_2_(II)X_n_] where L is a macrocyclic diphenolate tetra(amine) ligand, X=co‐ligand such as acetate, are very active and some of them show catalytic synergy.^[18]^ Many of these catalysts are effective for either carbon dioxide/epoxide or anhydride/epoxide ROCOP. For example, a heterodinuclear Mg(II)Zn(II) catalyst showed significantly greater activity in CHO/CO_2_ or PA ROCOP than equivalent Mg(II)Mg(II) or Zn(II)Zn(II) catalysts, providing evidence for intermetallic cooperativity.^[18g]^ In 2020, a Mg(II)Co(II) catalyst showed even higher rates in CHO/CO_2_ ROCOP and was also synergic (i. e., Mg(II)Co(II)≫Mg(II)Mg(II) or Co(II)Co(II)).^[19]^ Experimental analyses revealed that synergy arises from different roles for each metal in the rate‐determining step. The Mg(II) ion delivers a lower transition state entropy, whilst the Co(II) ion delivers a lower transition state enthalpy, compared with equivalent homodinuclear catalysts. These synergic catalysts merit further investigation as they provide an under‐explored means to increase catalytic rates. Nonetheless, not all hetero‐metallic complexes are synergic in these polymerization catalyses. For example, complexes of the same macrocycle coordinated to Zn(II)M, where M=Li(I), Na(I), K(I), Ca(II), Al(III), Ga(III) or In(III), were all much less active than the Zn(II)Zn(II) species.^[20]^ A dinucleating Schiff base ligand derived from *o*‐vanillin, also afforded catalysts showing similar reactivity trends Zn(II)Zn(II)>Zn(II)Ca(II)>Zn(II)Cd(II)≫Zn(II)Na(I).^[21]^ This prior work indicates that heterodinuclear combinations of Zn(II) with Group 1–3 metals are not synergic and, even in the case of Zn(II)Mg(II) complexes catalytic synergy depends upon the ancillary ligand and coordination chemistry.

To deepen understanding of dinuclear heteroallene/epoxide ROCOP catalysts, a series of heterodinuclear Mg(II)M(II) complexes, where M=first row transition metals from Cr(II) to Zn(II), were targeted. The goals of the research are: 1) To systematically investigate the influences of transition metals upon the overall catalytic performance; 2) To identify which metal combinations, if any, outperform the Mg(II)Mg(II) complex and 3) To understand whether activity trends directly translate between epoxide/anhydride and epoxide/carbon dioxide ROCOP. To ensure all trends are correctly interpreted and to allow comparisons with previously reported dinuclear catalysts, the same diphenolate tetraamine macrocyclic ancillary ligand should be used. The macrocycle pro‐ligand is efficiently synthesized in >60 % yield, using straightforward procedures.^[18c]^ First row transition metal ions are selected because the current best heterodinuclear catalysts, that is, Mg(II)Co(II) and Mg(II)Zn(II), both feature a transition metal.[^18f^, ^18g^, ^19^] The Mg(II) partner is selected due to its track record for high rates in carbon dioxide/epoxide ROCOP catalysis and for its abundance, light weight, low cost, lack of colour and low toxicity.^[22]^

## Results and Discussion

**Figure 2:**
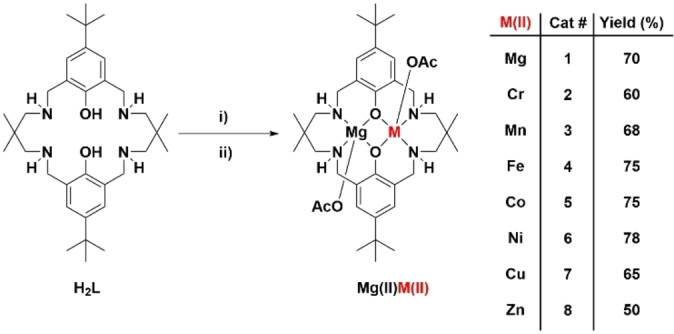
Synthesis and numbering scheme for Mg(II)M(II) catalysts **1–8**. Reagents and Conditions: i) Mg{N(Si(CH_3_)_3_)_2_}_2_, THF, 25 °C, 1 h. ii) M(OAc)_2_ (where M=Mg, Cr, Mn, Fe, Co, Ni, Cu or Zn), THF, 100 °C, 16 h.

Catalysts **1**–**8** were synthesized using the same macrocyclic pro‐ligand, H_2_L, by first reaction with Mg{N(Si(CH_3_)_3_)_2_}_2_, followed by the appropriate M(II) acetate, in THF at 100 °C and for 16 h (Figure 2). Complexes were isolated, after solvent removal and pentane washing to remove any residual amine, in moderate/high yields in all cases (>50 %). Complexes **1**–**8** were characterized by mass spectrometry (Figure S1–S8 in Supporting Information), cyclic voltammetry (Figure S9–S15) and infrared spectroscopy (Figure S16), where appropriate. All complexes were also characterized by elemental analysis and the combined data strongly suggest heterodinuclear complex formation.

Mass spectrometry (MALDI‐ToF) analysis confirms formation of complexes **2**–**7** and, in each case, the molecular cation was clearly observed, that is, [LMg(II)M(II)(OAc)]^+^ where M(II)=Cr(II) 685, Mn(II) 688, Fe(II) 689, Co(II) 692, Ni(II) 691 and Cu(II) 692 amu. The molecular ions show isotope distribution patterns consistent with predicted values (Figure S1–S8). In some cases, additional peaks corresponding to homodinuclear cations were also observed. These species are tentatively attributed to metal re‐distribution processes occurring during ionization since catalyst **8** (Mg(II)Zn(II)) shows such ions but its ^1^H NMR spectrum is free from any signals for the Zn(II)Zn(II) or Mg(II)Mg(II) complexes.

Complexes **1**–**7** were also characterized using cyclic voltammetry, used both to investigate the oxidation state(s) of the transition metal, M, and of the ligand (Figure S9–S15). These investigations were conducted using solutions of each complex dissolved in THF (0.1 M) and using [^n^Bu_4_N][PF_6_] (0.1 M) as the supporting electrolyte. All measurements were performed in a glovebox, under a nitrogen atmosphere, and the redox potentials are calibrated against the ferrocenium/ferrocene (Fc^+^/Fc) redox couple (assigned a value of 0.00 V). Complexes **1**–**7** each display a series of oxidations, at values above 0.39 V, which are attributed to phenolate radical oxidations, i. e. to ligand oxidation.[^19^, ^23^] Complex **2** shows an irreversible oxidation, at *E*_pc_=−0.35 V, assigned to Mg(II)Cr(II)/Mg(II)Cr(III)^+^; a reversible redox transition, at *E*_1/2_=−1.5 V, assigned to Mg(II)Cr(I)^−^/Mg(II)Cr(II); and an irreversible reduction, at *E*_pa_=−2.25 V, assigned Mg(II)Cr(I)^−^/Mg(II)Cr(0)^2−^ (Figure S10). These assignments correlate with typical redox potentials for other chromium complexes.^[24]^ Complex **3** shows an irreversible oxidation, at *E*_pc_=−0.10 V, for Mg(II)Mn(II)/Mg(II)Mn(III)^+^ and an irreversible reduction, at *E*_pa_=−0.75 V, assigned to Mg(II)Mn(II)/Mg(II)Mn(I)^−^, once again the transitions are assigned by analogy to similar manganese complexes (Figure S11).^[25]^ Complex **4**, exhibits two irreversible oxidations, at *E*_pc_=−0.5 V and 0.3 V, assigned to Mg(II)Fe(II)/Mg(II)Fe(III)^+^ and Mg(II)Fe(III)^+^/Mg(II)Fe(IV)^2+^, respectively, and an irreversible reduction, at *E*_pa_=−1.2 V, due to Mg(II)Fe(II)/Mg(II)Fe(I)^−^ (Figure S12).^[26]^ Complex **5** shows the same redox potentials as previously reported and is fully consistent with the formation of Mg(II)Co(II) (Figure S13).^[19]^ Complexes **6** and **7** did not show any clear M(II) oxidations, likely because these processes are obscured by the ligand centred phenolate oxidations (>0.4 V, Figure S14 and S15).^[27]^ Overall, the redox potentials are consistent with both the formation of and anticipated high electrochemical stability of Mg(II)M(II) complexes. Further, the data indicate that there are not significant quantities of contaminating homodinuclear complexes because, where these are present, two similar but different oxidations would be observed for each M(II) redox transition. For example, the Mg(II)Co(II) displays a single Co(II) to Co(III) oxidation at *E*_pa_=−0.06 V whereas the homodinuclear Co(II)Co(II) displayed two Co(II) to Co(III) oxidations at *E*_pa_=−0.06 V and *E*_1/2_=−0.10 V, respectively.^[19]^ All the complexes were also analysed using IR spectroscopy, and show characteristic signals for the NH (3298 cm^−1^) and acetate (ν_asym_=1590 cm^−1^ and ν_sym_=1440 cm^−1^, bending=924 cm^−1^) functionalities. Furthermore, there is a loss of the out‐of‐plane phenol OH deformation (δ_Ar−OH_=707 cm^−1^) when compared to the pro‐ligand confirming complex formation (Figure S16).

Complexes **1**–**8** were tested as catalysts for CO_2_/CHO ROCOP, using standard conditions, comprising of one atmosphere CO_2_ pressure, 1 : 4000 catalyst: cyclohexene oxide loading (i. e., 0.025 mol%), with 20 equivalents of *trans*‐1,2‐cyclohexanediol (CHD) as chain transfer agent and at 100 °C (Table 1).^[4b]^ Each polymerization was monitored using in situ IR spectroscopy; the change in absorption (increase) of characteristic polycarbonate resonances (1780, 1330 and 988 cm^−1^) were monitored over time. Each polymerization was analysed in triplicate, by independent runs, to ensure reproducible data. The average error in productivity, activity and rate coefficient values was ±5 %.

**Table 1:** CO_2_/CHO ROCOP data for catalysts **1–8** compared with selected high‐performance literature catalysts (See Figure 1 for reaction scheme).^[a]^

Entry	[LMg(II)M(II)(OAc)_2_] M, cat #	Conv. [%]^[b]^	CO_2_ [%]^[c]^	Polym. [%]^[d]^	TON^[e]^	TOF [h^−1^]^[f]^	*k*_p_ [mM^−1^ s^−1^]^[g]^	*k*_rel_^[h]^	*M*_n_ [kg mol^−1^] [Ð]^[i]^
1	Mg, **1**	57	>99	>99	2280	295 (±60)	9.7 (±0.2)	1.0	9.2 [1.15]
2	Cr, **2**	40	>99	>99	1600	103 (±19)	3.4 (±0.1)	0.4	4.0 [1.15]
3	Mn, **3**	76	>99	90	3040	362 (±31)	11.9 (±0.2)	1.2	4.1 [1.23]
4	Fe, **4**	59	>99	>99	2360	1071 (±33)	34.7 (±0.3)	3.6	8.4 [1.11]
5	Co, **5**	59	>99	>99	2360	1056 (±19)	34.7 (±0.1)	3.6	8.0 [1.11]
6	Ni, **6**	60	>99	>99	2400	632 (±34)	20.7 (±0.2)	2.2	6.3 [1.16]
7	Cu, **7**	47	>99	94	1880	136 (±3)	4.5 (±0.1)	0.5	6.5 [1/15]
8	Zn, **8**	37	>99	>99	1480	506 (±16)	14.4 (±0.1)	1.5	5.0 [1.17]
9^[j][28]^	[(proline)Zn_2_]	*n.r*	>99	>99	1684	149	–	–	*n.r*
10^[k][17d]^	[(trisalen)Zn_3_Ce(OAc)_3_]	45	>99	>99	900	300	–	–	15.0 [1.20]
11^[l][29]^	[(L′)Zn_2_Na(I(OAc)]	49	86	97	1960	478	–	–	5.6 [1.29]
12^[m][30]^	[(salen[NR_3_]^+^)Co(DNP)_2_]	26	>99	>99	1315	263	–	–	48.2 [1.12]
13^[n][31]^	[(L′′)Ni_2_(OBz)_2_(MeOH)]	18	>99	>99	288	96	–	–	8.7 [1.21]

[a] Reaction conditions: [cat]_0_:[CHD]_0_:[CHO]_0_=1 : 20 : 4000, 0.025 mol% catalyst loading (2.44 mM), 20 equivalents *trans*‐1,2‐cyclohexanediol, CHD, (48.8 mM), neat CHO (6 mL, 9.9 M), 1 bar CO_2_, 100 °C. [b] Expressed as a percentage of epoxide conversion against the theoretical maximum (100 %). [c] Expressed as a percentage of CO_2_ uptake against ether linkage formation. [d] Expressed as a percentage of polymer selectivity against cyclic carbonate formation. [e] Turn over number (TON)=number of moles of monomer converted/number of moles of catalyst. [f] Turn over frequency (TOF)=TON/hour. [g] *k*_p_=polymerization rate coefficient=*k*_obs_/[cat]^1^ where *k*_obs_ is calculated from the gradient of the semi logarithmic plot of ln([CHO]_t_/[CHO]_0_) vs. time (s). [h] *k*_rel_=relative polymerization rate coefficient=*k*_p(MMg)_/*k*_p(MgMg)_. [i] Determined by SEC analysis, in THF, calibrated with narrow‐*M*_n_ polystyrene standards; dispersity values in parentheses. [j] Reaction conditions: 0.1 mol% catalyst loading, 1 bar CO_2_, 80 °C. [k] Reaction conditions: 0.05 mol% catalyst loading, neat CHO (9.9 M), 3 bar CO_2_, 100 °C. [l] Reaction conditions: 0.025 mol%, 20 equiv. CHD, 100 °C, 1 bar CO_2_. [m] Reaction conditions: 0.02 mol% catalyst loading, 1 bar CO_2_, 50 °C. [n] Reaction conditions: 0.0625 mol% catalyst loading, neat CHO (100 mmol), 1 bar CO_2_, 100 °C. *n.r*=not reported. For illustrations of the literature catalyst structures, and explanations of the ancillary ligand abbreviations used, see Figure S17.

For each polymerization, a pseudo first order rate coefficient, *k*_obs_ (s^−1^), was determined by an initial rates method (i. e., by monitoring the conversion vs. time data over the range 5–20 % conversion, Figure S18–S24). It was previously shown that monitoring the initial rate coefficient, that is, 5–20 % conversion, showed identical polymerization rates coefficients (*k*_obs_), within error, to those monitored by an integrated rate methods, that is, from 5–80 % conversion.[^19^, ^32^] The polymerization propagation rate coefficient (*k*_p_) was determined from the pseudo first order rate coefficient (*k*_obs_) by correcting for the relevant catalyst concentration (N.B, this treatment assumes all catalysts show the same first order in catalyst concentration in the rate law which was experimentally determined only for catalysts **5** and **8**).[^18f^, ^19^]

Catalysts **1**–**8** all showed good performances with most having quantitative carbon dioxide uptake into the polymer (>99 %) and forming polycarbonates free from any ether linkages (^1^H NMR spectroscopy, Figure S26). Most catalysts show high polymer selectivity (>99 %), except for complexes **3** and **7** which formed up to 10 % *trans*‐cyclic carbonate (Figure S25). In all cases, the polycarbonate (PCHC) molar mass values, measured by SEC, are close to the theoretical values (*M*_*n(theory)*_=8.0 kg mol^−1^) and have monomodal, narrow dispersity distributions (Ð <1.25) (Table 1). End‐group analysis (MALDI‐ToF) showed high selectivity for *α, ω*‐hydroyl telechelic polyols which are initiated from the chain transfer agent (*trans*‐1,2‐cyclohexane diol) (Figure S26). All the new complexes are effective catalysts but there are metal dependent differences in productivity and activity. The best catalysts are complexes **4** and **5**_,_ featuring Fe(II) or Co(II), respectively. Catalyst **5** shows an activity of 1056±19 h^−1^ (*k*_p_=34.7±0.1 mM^−1^ s^−1^) and remains the fastest low pressure (1 bar) catalyst reported to date.^[19]^ Complex **4** is also highly active, having an equivalent turn‐over‐frequency of 1071±33 h^−1^ (*k*_p_=34.7±0.3 mM^−1^ s^−1^). The results for catalyst **4** set a new upper activity bound for low pressure iron catalysts in this field.^[33]^ Complex **4** may offer advantages over catalyst **5** in the longer term since iron is highly abundant (56 000 ppm) and generally has low toxicity, whereas cobalt is the least abundant first row transition metal (25 ppm) and its compounds can show toxicity.^[34]^

Iron‐based catalysts are still quite rare in the ring opening copolymerization of carbon dioxide with epoxides.^[33]^ In 2011, our team reported the first iron catalyst for the ring opening copolymerization of CO_2_ with cyclohexene oxide.^[35]^ Activity was low at 1 bar carbon dioxide pressure (TOF=6 h^−1^, 0.1 mol%, 80 °C) but improved at 10 bar pressure (TOF=107 h^−1^, 0.01 mol%, 80 °C).^[35]^ Iron complexes coordinated by ligands with a strong precedent in this catalysis, such as salens,^[36]^ salans^[37]^ and porphyrins,^[38]^ all form cyclic carbonates rather than the desired polymers. Other iron catalysts for carbon dioxide/epoxide copolymerization apply corrole,^[39]^ tris‐^[40]^ or bis‐phenolate,^[41]^ ligands and generally require high catalyst loadings (>0.1 mol%), high carbon dioxide pressure (>10 bar) and co‐catalysts. For example, in 2013, Kleij and Pescarmona reported an iron(III)(amino tris(phenolate))/PPNCl catalyst system which operated under low carbon dioxide pressures (3 bar) and showed moderate activity (TOF=29 h^−1^, 0.1 mol% catalyst, 0.05 mol% PPNCl, 60 °C). Compared with these prior Fe(III) catalysts, complex **4** is 37 x more active (TOF=1071 h^−1^, 0.025 mol%, 100 °C), operates at 4 x lower catalyst loadings and does not need any co‐catalyst.

Considering the next most active catalyst in the series, Mg(II)Ni(II) catalyst, **6**, and comparing it to other known Ni(II) catalysts also reveals some promising features.[^31^, ^42^] Ko and co‐workers pioneered Ni(II) catalysts, reporting a series of high activity di‐Ni(II) complexes, coordinated by modified benzotriazole Schiff‐base ligands (TOF=9600 h^−1^, 0.01 mol%, 140 °C, 21 bar).[^31^, ^42b^, ^43^] The catalysts also perform well at 1 bar CO_2_ pressure, for example showing a TOF of 96 h^−1^ (0.06 mol%, 100 °C, 1 bar).^[42b]^ Comparably, the Mg(II)Ni(II) catalyst **6** shows a 6 x higher activity under similar conditions (TOF=632 h^−1^, 0.02 mol %, 100 °C, 1 bar). The next most active catalyst in the series is the Mn(II)Mg(II) which not only shows a good performance but also represents the first example of a Mn(II) catalyst in this field. There is precedent for Mn(III)/PPNCl catalyst systems, for example complexes featuring porphyrin,^[44]^ corrole,^[45]^ *N*,*N*‐bis(trifluoroacetylacetone)‐1,2‐ethylenediimine or *N,N′*‐bis(acetylacetone)‐1,2‐ethylendiamine,^[46]^ or salen ligands.^[44a]^ Generally, the performances of Mn(III) catalysts are quite modest and Darensbourg and co‐workers attributed it to a low binding affinity of 5‐coordinate Mn(III) model complexes with epoxide/anions.^[46b]^ It was proposed that the Mn(III) complexes are inert and that this was responsible for the low polymerization activity compared to Cr(III) or Co(III) analogues.^[46b]^ Catalyst **3** is not only the first Mn(II)‐catalyst, but it also demonstrates surprisingly high activity at only 1 bar CO_2_ pressure (TOF=362 h^−1^, 0.025 mol%, 100 °C). Such an activity comfortably surpasses (100 x) the next most active Mn(III)‐porphyrin complex under similar conditions (TOF=3 h^−1^, 0.2 mol%, 1 bar, 80 °C). The higher activity appears to be linked to the presence of the neighbouring Mg(II) ion. It is proposed that magnesium coordinates the epoxide and thereby overcome the prior binding strength limitations of Mn(III) complexes. This result highlights both the importance of the s‐block ion and the advantages of the dinuclear catalysts where metals adopt to distinctive roles in the transition state for epoxide ring opening (rate‐determining step).^[19]^ Overall, most of the Mg(II)M(II) complexes showed better rates than the homodinuclear complex **1**, except for the complexes featuring Cr(II) (*k*_p_=3.4±0.1 mM^−1^ s^−1^) and Cu(II) (*k*_p_=4.5±0.1 mM^−1^ s^−1^). The performance of the Cr(II) complex is, perhaps, rather surprising since many Cr(III) catalysts for CO_2_/epoxide catalysis are reported.^[47]^ The low activities of both Cr(II) and Cu(II) heterodinuclear catalysts may arise from a stabilizing Jahn‐Teller distortion resulting in axial bond compression, thus increasing the M−O_2_COR bond strength and reducing the lability of the M−O_2_COR species in epoxide ring‐opening.

The most active Mg(II)M(II) heterodinuclear catalysts perform well when compared to high performance catalysts in this field, especially compared with other low pressure (1 bar) carbon dioxide copolymerization catalysts (Table 1, Entries 9–13). Specifically, catalysts **4** and **5** show an 11 x greater activity than a leading di‐Ni(II) complex (Table 1 Entry 13), 5 x greater activity than a di‐Zn(II) complex (Table 1 Entry 9), 3 x greater activity than both a tetrametallic Zn_3_Ce catalyst (Table 1 Entry 10) or an active ammonium tethered Co(III) catalyst (Table 1 Entry 12), and 2 x higher activity than a recently reported trimetallic Zn(II)_2_Na catalyst (Table 1 Entry 11).[^17d^, ^28^, ^29^, ^30^, ^31^] Direct comparisons are challenged by the range of reaction conditions being used by different researchers but overall these catalysts are amongst the best low pressure carbon dioxide polymerization catalysts. They also show equivalent activity to a recently reported multi‐metallic catalyst, [(trisalen)Co(II)_3_La(III)(OAc)_3_], (TOF=1375 h^−1^). The Co(II)_3_La(III) catalyst was tested at both higher CO_2_ pressure (20 bar) and temperature (130 °C); these conditions highlight the remarkable activities of **4** and **5**. When applied under closer conditions to those used in this work (CO_2_ pressure 5 bar), the Co(II)_3_La(III) catalyst shows only low polymer selectivity (40 %).^[17e]^

**Figure 3:**
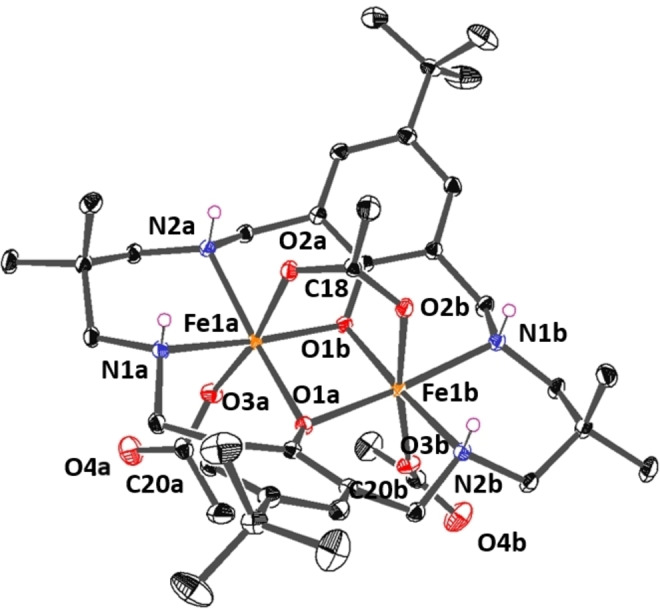
ORTEP representations of the molecular structure of an Fe(II)Fe(III) complex obtained by single‐crystal X‐ray diffraction experiments. Complex disorder issues and H‐atoms (exception of NH) are omitted, for clarity, and the thermal ellipsoids are represented at 40 % probability. For selected bond lengths (Å) and angles (°) see Table S1.

To better understand the performance of catalyst **4**, the homodinuclear Fe(II)Fe(II) complex was targeted. The macrocyclic pro‐ligand was reacted with two equivalents of iron(II) acetate and a purple complex was isolated. Cyclic voltammetry indicated its speciation as Fe(II)Fe(III) (Figure S27). This speciation was suggested by a single reversible redox couple at *E*_1/2_=−0.5 V, attributed to Fe(II)Fe(III)/Fe(III)Fe(III)^+^ and an irreversible reduction, attributed to Fe(II)Fe(III)/Fe(II)Fe(II)^−^, at *E*_pa_=−1.75 V. These findings are indicative of a mixed oxidation state Fe(II)Fe(III) complex since there is only one Fe based oxidation, whilst two are expected for a complex of oxidation state Fe(II)Fe(II). The mixed oxidation states were also supported by single‐crystal X‐ray diffraction experiments; single crystals of the di‐iron complex were isolated by pentane diffusion into a saturated THF solution of the complex (Figure 3, Figure S28). In the solid state, Fe(II)Fe(III) adopts a monomeric structure with each iron centre having a hexa‐coordinate, octahedral geometry. The iron centres are coordinated by the ligand, which adopts a planar configuration, and by two acetate co‐ligands which coordinate perpendicular to the ligand plane. One acetate group bridges (μ_2_‐OAc) the iron centres, whilst the other is coordinated to a single iron (μ_1_‐OAc). The iron centres are crystallographically identical, related by a C_2_‐axis of rotation, and the terminal and bridging iron−acetate bonds (Fe_1_−O2a or O3a) measure 1.9644(14) Å and 2.0946(13) Å, respectively. The two acetate carbon−oxygen bonds in the bridging ligand, C18−O2a and C18−O2b are identical (1.2593(17) Å) which indicates complete electronic delocalization across the acetate and a hypothetical bond order of 1.5, whereas the terminally coordinated acetate ligand shows the expected different bond lengths, C20−O3a (1.275(2) Å) and C20−O4a (1.226(3) Å). The symmetrical iron centre coordination environments suggest charge delocalization between the two centres, rather than one iron centre having the formal oxidation state +2 and the other +3. The intermetallic distance is 2.8999(5) Å, similar to other complexes of this macrocycle and consistent with the expected distances for cooperative catalysis.[^20^, ^32^, ^48^]

The Fe(II)Fe(III) complex is tentatively proposed to form by oxidation of the Fe(II)Fe(II) complex with the acetic acid by‐product (formed from ligand deprotonation), perhaps assisted by residual oxygen dissolved in the solvent (Scheme S1). The Fe(II)Fe(II) complex was successfully synthesised when ligand deprotonation was achieved by reacting with K{N(Si(CH_3_)_3_)_2_} to form a bis‐potassium complex in situ, followed by the addition of iron(II) acetate. The reaction resulted in the precipitation of potassium acetate which was removed and, after washing and drying, the product was isolated as a dark red powder (Scheme S1). The Fe(II)Fe(II) speciation was confirmed by cyclic voltammetry (Figure S29) and mass spectrometry (Figure S30); purity was determined by elemental analysis. In contrast to Fe(II)Fe(III), the cyclic voltammogram of Fe(II)Fe(II) shows two reversible redox couples assigned to Fe(II)Fe(II)/Fe(II)Fe(III)^+^ and Fe(II)Fe(III)^+^/Fe(III)Fe(III)^2+^ oxidations, at *E*_1/2_=0.25 V and −1.10 V, respectively (Figure S29). Unfortunately, neither of the di‐iron complexes, that is, Fe(II)Fe(II) and Fe(II)Fe(III), were active in CO_2_/CHO ROCOP at 1 bar pressure. This is rather surprising as both the analogous Co(II)Co(II) and Co(II)Co(III) complexes show good activities (TOF=410 h^−1^ and 500 h^−1^, respectively) under similar conditions (0.1 mol%, 100 °C, 1 bar CO_2_).^[49]^ Overall, this result further underscores the importance of the Mg(II) ion in catalyst **4** in accelerating rates and providing a site for epoxide coordination to allow for low pressure activity.

Frequently, catalysts active for the ring opening copolymerization of carbon dioxide/epoxides are also active for anhydride/epoxide ROCOP. Here, the activity and selectivity data for these catalysts in the NA/CHO ROCOP were determined. Although phthalic anhydride is the more typical anhydride used to compare new catalysts, norbornene anhydride is useful in providing an internal alkene for post‐functionalization experiments, and is commercially available.^[50]^ Recently, the selective post‐functionalization of these internal alkene functional groups (on the anhydride monomer), via thiol‐ene reactions, delivered amphiphilic polyesters able to self‐assemble in solution.^[51]^

Complexes **1**–**8** were tested for NA/CHO ROCOP at a catalyst loading of 0.05 mol % (4.88 mM) with 20 equivalents CHD, in neat CHO (9.9 M), at 100 °C (Table 2). All reactions were monitored using *in situ* IR spectroscopy and kinetics were determined by measuring the increase in the absorbance of polyesters (1750 cm^−1^) against time. For each polymerization the observed rate coefficient, *k′*_obs_ (s^−1^), was determined as the gradient of linear fits to anhydride absorbance versus time plots across the whole (1 ‐ >99 %) conversion range (Figure S32–S38). Catalysts **1**–**8** displayed excellent polyester selectivity (>99 %) with no ether linkages being observed in the resulting polyesters, as determined by ^1^H NMR spectroscopy. All polymerizations resulted in monomodal polymer molar mass distributions with narrow dispersity, as measured by SEC. All molar masses measured were close to the theoretical (∼1.3 kg mol^−1^).

**Table 2:** NA/CHO ROCOP data using catalysts **1**–**8** and compared against leading catalysts from the literature (See Figure 1 for reaction scheme).^[a]^

Entry	[LMg(II)M(II)(OAc)_2_] M, cat #	Conv. [%]^[b]^	Polymer [%]^[c]^	TON^[d]^	TOF [h^−1^]^[e]^	*k*_p_′ [mM^−1^ s^−1^]^[f]^	*k*_rel_′^[g]^	*M*_n_ [kg mol^−1^] [Ð]^[h]^
1	Mg, **1**	>99	>99	100	71 (±4)	8.7 (±0.5)	1.0	1.6 [1.15]
2	Cr, **2**	>99	>99	100	54 (±3)	6.6 (±0.4)	0.8	*n.d*
3	Mn, **3**	>99	>99	100	272 (±13)	33.5 (±1.6)	3.9	1.4 [1.14]
4	Fe, **4**	>99	>99	100	109 (±5)	13.4 (±0.6)	1.5	1.3 [1.15]
5	Co, **5**	>99	>99	100	610 (±31)	75.2 (±3.8)	8.6	1.5 [1.14]
6	Ni, **6**	>99	>99	100	244 (±12)	30.1 (±1.5)	3.5	1.4 [1.15]
7	Cu, **7**	>99	>99	100	170 (±9)	21.2 (±1.1)	2.4	1.2 [1.15]
8	Zn, **8**	>99	>99	100	186 (±10)	23.0 (±1.2)	2.6	1.3 [1.14]
9^[i][52]^	(ONSO)CrCl/PPNCl	97	>99	243	360	–	–	11.9 [1.28]
10^[j][50]^	(salophen)CrCl/DMAP	97	>99	243	49	–	–	3.0 [1.12]
11^[k][13a]^	(salen[CyPr]^+^)AlCl_2_	51	>99	204	34	–	–	8.2 [1.24]
12^[l][53]^	(*o*‐van)AlK(OAc)_2_	33	>99	133	266	–	–	4.5 [1.10]
13^[m]^	Co	>99	>99	100	666 (±21)		–	2.2 [1.14]

[a] Reaction conditions: [cat]_0_:[CHD]_0_:[NA]_0_:[CHO]_0_=1 : 20 : 100 : 2000; 0.05 mol% catalyst loading (4.88 mM), 20 equivalents *trans*‐1,2‐cyclohexendiol (97.6 mM), neat CHO (3.2 mL, 9.9 M), 100 °C. [b] Expressed as a percentage of epoxide conversion against the theoretical maximum (100 %). [c] Expressed as a percentage of polymer selectivity against cyclic carbonate formation. [d] Turn over number (TON)=number of moles of monomer converted/number of moles of catalyst. [e] Turn over frequency (TOF)=TON/hour. [f] *k*_p_=rate coefficient=*k*_obs_/[cat]^1^ where *k*_obs_ is calculated from the gradient of the plot of [NA] vs. time (s). [g] *k*_rel_=relative rate coefficient=*k*_p(MgM)_/*k*_p(MgMg_). [h] Determined by SEC analysis, in THF, calibrated with narrow‐*M*_n_ polystyrene standard; dispersity values in parentheses. [i] Reaction conditions: [cat]_0_/[PPNCl]_0_:[NA]_0_:[CHO]_0_=1 : 1 : 250 : 250 in toluene (2 mL), 110 °C. [j] Reaction conditions: [cat]_0_:[DMAP]_0_:[NA]_0_:[CHO]_0_=1 : 1 : 250 : 250 in toluene, 110 °C. [k] Reaction conditions: [cat]_0_:[NA]_0_:[CHO]_0_=1 : 400 : 2000, 0.05 mol % catalyst loading, neat CHO (9.9 M), 60 °C. [l] Reaction conditions: [cat]_0_:[NA]_0_;[CHO]_0_=1 : 400 : 2000, 0.05 mol %, neat CHO (9.9 M), 100 °C. [m] Reaction with Phthalic Anhydride (PA). For reported catalyst structures, entries 9–13, see Figure S31.

All catalysts were active and broadly the same activity trend was observed as for carbon dioxide/cyclohexene oxide ROCOP. For example, the Mg(II)Co(II) catalyst showed the highest activity (TOF=610±31 h^−1^, *k*_p_′=75.2±3.8 mM^−1^ s^−1^) followed by Mg(II)Mn(II) (TOF=272±13 h^−1^), Mg(II)Ni(II) (TOF=244±12 h^−1^) and Mg(II)Zn(II) (TOF=186±10 h^−1^). In common with the carbon dioxide copolymerization catalysis, the Mg(II)Cr(II) catalyst performs poorly (TOF=54±3 h^−1^). In contrast, the Mg(II)Cu(II) catalyst performs surprisingly well (TOF=170±9 h^−1^) and the Mg(II)Fe(II) under‐performs compared with its equivalent activity in carbon dioxide copolymerization (TOF=109±5 h^−1^).

Compared with other leading CHO/NA ROCOP catalysts, complex **5** shows good activity. For example, it has 12 x higher activity than the commonly implemented [(salphen)Cr(III)Cl]/PPNCl catalyst system (TOF=49 h^−1^, 0.4 mol %, 110 °C) and equivalent activity, at ten times lower loading, to a sulfur modified salen, [(ONSO)Cr(III)Cl]/PPNCl, catalyst system (TOF=360 h^−1^, 0.4 mol%, 110 °C) (Table 2 Entry 9 and 10).[^50^, ^52^] Since **5** is active without co‐catalyst it can be applied at lower loading than the metal salen based systems. So far, there is only one tethered salen‐co‐catalyst system reported for the copolymerization of NA/CHO (Table 2 Entry 11). Although catalyst **5** appears to be more active than the [(salen[CyPr]^+^)Al(III)Cl] catalyst it is applied at higher temperature (100 °C versus 60 °C) which prevents fair comparison.^[13a]^ Very recently, we reported a heterodinuclear Al(III)K(I) catalyst for NA/CHO ROCOP, [(*o*‐van)Al(III)K(I)(OAc)_2_], and in comparison **5** is twice as active under equivalent conditions (TOF=266 h^−1^, 0.05 mol%, 100 °C).^[53]^

Because phthalic anhydride/CHO ROCOP is a more commonly used monomer, catalyst **5** was also tested using it under otherwise equivalent conditions ([Cat]_0_:[PA]_0_:[CHO]_0_=1 : 100 : 2000, 0.05 mol%, 100 °C). Catalyst **5** performs well compared to other dinuclear catalysts, such as [(*o*‐van)Zn(II)_2_(OAc)_2_ (TOF=198 h^−1^, 0.125 mol%, 100 °C) and to Zn(II)Mg(II) catalysts.[^21^, ^54^] Although catalyst **5** showed slightly higher activity compared with [(*o*‐van)Al(III)K(I)(OAc)_2_] for NA/CHO ROCOP, it underperforms against this same catalyst for PA/CHO with the Al(III) catalyst showing very high activities (TOF=1072 h^−1^, 0.05 mol%, 100 °C).^[53]^ Other highly active multimetallic catalysts were all used with the co‐catalyst PPNCl, and include [(trisphenolate)Fe(III)]_2_ (TOF=588 h^−1^, 100 °C),^[41c]^ [(salen)Al(III)Cl]_2_ (TOF=750 h^−1^, 50 °C)^[55]^ and [(salen)Cr(III)Cl]_3_ (TOF=10 620 h^−1^, 0.001 mol%, 100 °C).^[56]^ These literature catalysts are excellent showing the highest activities for anhydride/epoxide ROCOP. In comparison, organocatalyst systems show lower activities, with typical TOF values being below 300 h^−1^ but may have advantages in terms of ease of use/commercial availability. For example, catalysts based on bases like ^t^BuP_1_ (TOF=48 h^−1^, 100 °C),^[57]^ BEt_3_/DBU (TOF=27 h^−1^, 60 °C),^[58]^ BPh_3_/PPNCl (TOF=200 h^−1^, 0.2 mol %, 130 °C)^[59]^ or (9‐BBN)B‐(C_4_H_10_)‐NMe_2_^n^Bu (TOF=258 h^−1^, 0.25 mol%, 120 °C).^[60]^

**Figure 4:**
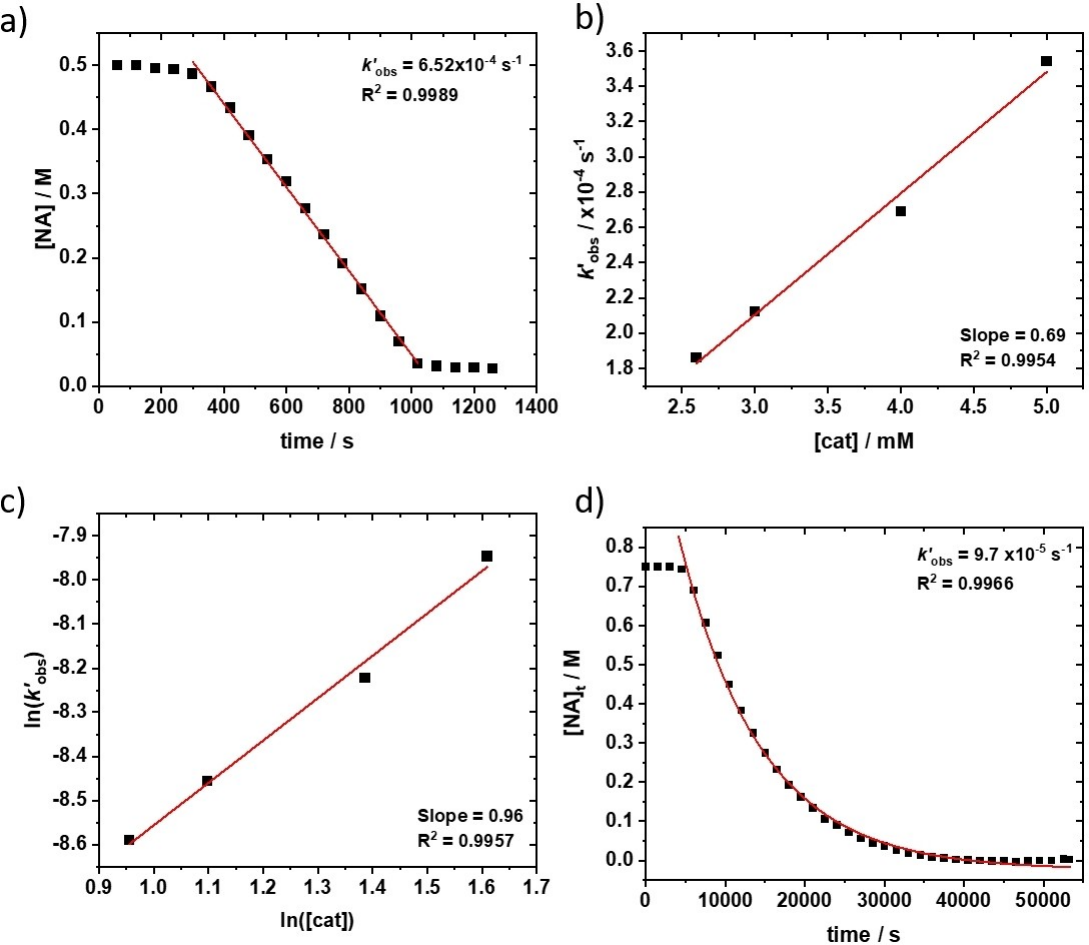
a) Reaction kinetic plots to determine the order with respect to a) [PA] (0^th^ order) b) and c) [Catalyst] and d) [CHO]. a) Reaction conditions: [cat]_0_:[CHD]_0_: [NA]_0_:[CHO]_0_=1 : 20 : 100 : 1000, [cat]_0_=10 mM, 100 °C. b)and c) Reaction conditions: [cat]_x‐y_:[CHD]_0_:[NA]_0_:[CHO]_0_=1–2 : 20 : 100 : 2000, [cat] _x‐y_=2.5 mM (x)–5 mM (y), 100 °C. d) [cat]_0_:[CHD]_0_:[NA]_0_:[CHO]_0_=1 : 20 : 180 : 150, [cat]_0_=5 mM in Tol (total volume 1.6 mL), 100 °C.

Since **5** shows the best performances of the series, its rate law was investigated for the ring opening copolymerization of anhydride/epoxide. Polymerizations were monitored using in situ infrared spectroscopy, following the stretching frequencies for anhydride (1820 cm^−1^) and polyester (1784 cm^−1^). Monomer conversions were also benchmarked using NMR spectroscopy, with internal standards, by comparing the integrals for NA (6.25 ppm) and polyester (4.5–4.9 ppm). To determine the order in anhydride concentration, two reactions were compared with a ten‐fold difference in NA concentrations ([cat]_0_:[CHD]_0_:[NA]_0_:[CHO]_0_=1 : 20 : 100 : 2000, [cat]=5 mM and [NA]=0.5 M, neat CHO (9.9 M), 100 °C). In each polymerization there was a linear decrease in NA concentration with time over the entire conversion range (0–100 %); strongly indicating a zeroth order in anhydride concentration (Figure 4a). This finding is consistent with the results for carbon dioxide/CHO ROCOP, where a zeroth order was also observed for the heteroallene. The order in catalyst concentration was determined from the pseudo rate coefficients (*k*_obs_), determined by linear fits to concentration versus time plots from a series of reactions with catalyst **5** concentrations from 2 mM–5 mM ([CHO]=9.9 M, [NA]=0.5 M, 100 °C). A linear relationship between *k*_obs_ and catalyst concentration was observed with a gradient close to 1 for the logarithmic plots – both findings are consistent with a first order in catalyst concentration (Figure 4b, c). To determine the order in epoxide concentration, an integrated rate treatment to a reaction conducted in toluene was conducted ([cat]_0_=5 mM, [NA]_0_=0.9 M, [CHO]_0_=0.75 M, toluene (1.5 mL)). An exponential fit to the data was applied (*k′*_obs_=9.7×10^−5^ s^−1^, R^2^=0.9966) across the conversion range (0–100 %), indicative of a first order dependence on epoxide concentration. The overall rate law can be summarized as [Eq. 1]:

(1) $$rate=\;k_{p}^{{}^{'}}{\left[cat\right]}^{1}{\left[epoxide\right]}^{1}{\left[anhydride\right]}^{0}$$

**Figure 5:**
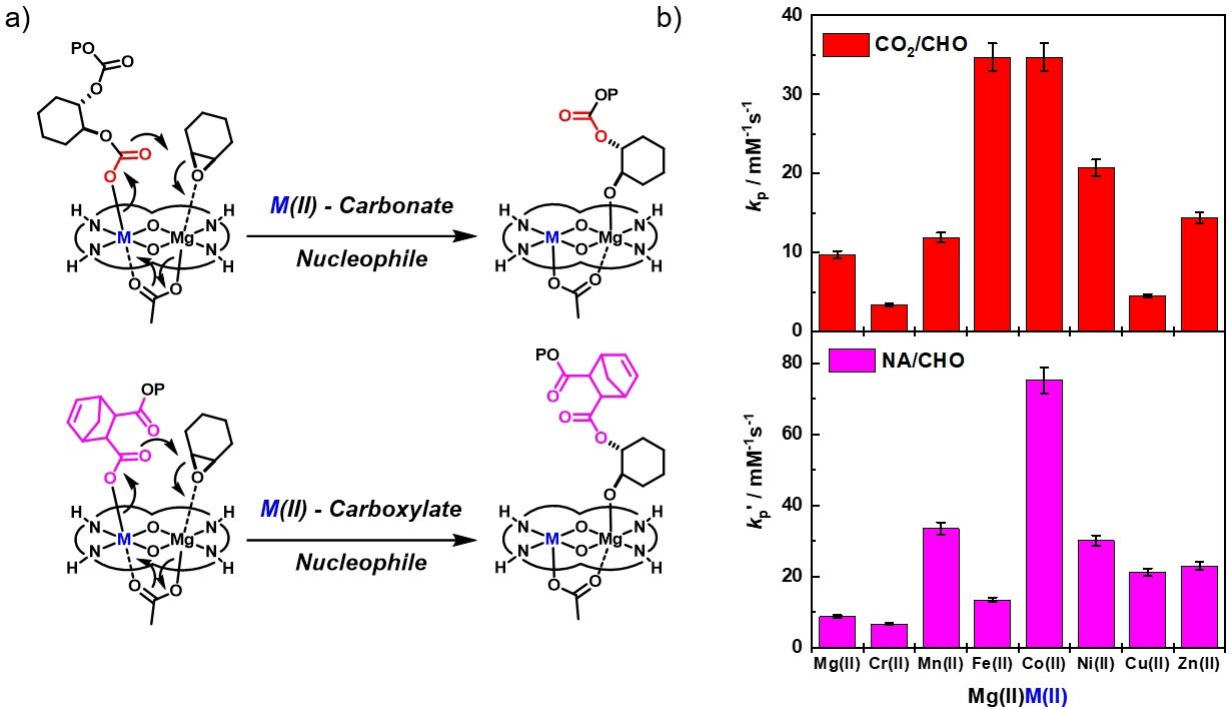
a) Illustration of the rate determining reactions in each polymerization with the Mg(II) coordinated cyclohexene oxide being ring‐opened by the M(II)‐carbonate or M(II)‐carboxylate nucleophile, respectively. b) Plots of the polymerization propagation rate coefficients determined for the ring opening copolymerization of CO_2_/CHO or NA/CHO using Mg(II)M(II) catalysts **1–8**.

The rate law indicates that the rate determining step is likely to be similar for the two polymerizations and involves cyclohexene oxide ring‐opening by the transition metal‐carbonate or transition metal‐carboxylate, respectively (Figure 5a). When comparing the ROCOP activity trends for the two polymerizations the similarities are apparent as one might expect for reactions involving a similar rate limiting step (Figure 5a). The CHO/CO_2_ ROCOP activity, in particular, shows a volcano trend against the first‐row transition metals, with the highest activity resulting from metals in the middle of the series, that is, Fe(II) and Co(II) (Figure 5b). The CHO/NA ROCOP catalysis shows a similar, but less pronounced, trend with activity being highest in the middle of the series but there is an unexpectedly low activity for catalyst **4**, Mg(II)Fe(II) (Figure 5b). The low activity is tentatively attributed to catalyst instability with respect to oxidation. Given the prior precedent for the oxidation of Fe(II) coordinated by this ligand using residual carboxylic acids and the likelihood that low levels of di‐carboxylic acid may be present as contaminants in the anhydride, the poor activity likely arises from catalyst decomposition by Fe(II) oxidation. The most active catalyst in both series is Mg(II)Co(II) and its absolute performance is excellent when compared against other catalysts in this field.

There is also no single correlation between activity and transition metal Lewis acidity, oxophilicity, bond dissociation energy (Figure S39), water exchange rate constants or hydrolysis constants (Figure S40); these findings are perhaps unsurprising since the reaction rate limiting step does not obviously depend upon a single factor.^[61]^ Nozaki, Mashima and co‐workers studied the influences of different lanthanide metals (M(III)=La, Ce, Pr, Nd, Eu or Gd) as part of tetranuclear catalyst of the form [LCo(II)_3_M(III)(OAc)_3_] – the work also failed to find clear correlations between activity values and ionic radius, bond dissociation energy, hydrolysis constant or water‐exchange rate constants.^[17e]^

One useful outcome from this structure‐activity study is the benefits of catalysts featuring Fe(II) or Co(II) combined with Mg(II), as well as the potential for catalysts using Mn(II) and Ni(II). It seems that mid‐period transition metals are most able to counterbalance the mechanistic requirements for both Lewis acidity and nucleophilic M−O_2_CR bonds, they also have ionic radii that can be accommodated within planar ligand coordination environments. This work suggests that further investigation of Fe(II) catalysts is warranted and strategies to exploit s‐block M(I/II)Fe(II/III) synergy in this catalysis should be explored. Being able to replace rarer transition metals, such as Co(II/III), with Fe(II/III) could offer some obvious advantages. It's also important to emphasise that Co(III) catalysts are very common to the carbon dioxide/epoxide ROCOP catalysis field, but both Co(II) and Fe(II) catalysts are under‐developed. Recently, several other high‐performance hetero‐multimetallic catalysts were reported; investigation of other heterodinuclear catalysts combining iron with sodium, potassium or magnesium is recommended.[^17a^, ^17b^, ^17d^, ^17e^, ^29^] There are many known di‐zinc catalysts and some of the ligands may allow access to Fe(II)Mg(II) complexes.[^16b^, ^62^] Recently, we reported high activity Co(III)K(I) catalysts for carbon dioxide/propylene oxide ROCOP, these structures could also be investigated for Fe(III), Mn(III) or Ni(III) coordination chemistry.^[32]^ Given that the catalyst reactivity trend is similar for ROCOP reactions, the best catalysts could also be used in switchable polymerizations and cyclic ester/carbonate ring‐opening polymerizations.[^9a^, ^18e^, ^32^, ^63^]

## Conclusions

The synthesis, characterization, and polymerization catalytic activity of a series of heterodinuclear complexes, combining Mg(II)M(II) (M=Cr(II), Mn(II), Fe(II), Ni(II) and Cu(II)) was reported. The catalysts were all active for both carbon dioxide/cyclohexene oxide and endo‐norbornene anhydride/cyclohexene oxide ring opening copolymerization; all catalysts showed high selectivity and polymerization control. The most active catalysts feature transition metals from the middle of the period, specifically Mg(II)Co(II) and Mg(II)Fe(II). These heterodinuclear catalysts outperform many other literature catalysts, particularly under low pressure conditions and function well for both polyester and ‐carbonate polymerizations. It remains challenging to predict metal synergy and undoubtedly more investigations are required, but these data implicate a fine balance between metal carbonate bond strength, lability, intermetallic distance and metal Lewis acidity as important parameters in synergic catalysis. There are clear benefits to substituting expensive and rare transition metals with inexpensive, abundant and low toxicity metals such as magnesium and iron; future work targeting these and other s‐block metal combinations is on‐going.

## Experimental Section

Experimental and characterization details can be found in the Supporting Information.

Deposition Numbers 2108011 (for **Fe(II)Fe(III)**) contains the supplementary crystallographic data for this paper. These data are provided free of charge by the joint Cambridge Crystallographic Data Centre and Fachinformationszentrum Karlsruhe Access Structures service www.ccdc.cam.ac.uk/structures.

## Author Contributions

NVR carried out all complex synthesis and characterization along with all CO_2_‐related experiments. GR carried out all NA‐related experiments. CBD collected and solved the Fe(II)Fe(III) X‐ray structure. ACD provided laboratory supervision and wrote the manuscript. CKW supervised all the research, wrote the manuscript and secured the research funding.

1

## Supporting information

As a service to our authors and readers, this journal provides supporting information supplied by the authors. Such materials are peer reviewed and may be re‐organized for online delivery, but are not copy‐edited or typeset. Technical support issues arising from supporting information (other than missing files) should be addressed to the authors.

Supporting Information

## Data Availability

The data that support the findings of this study are available from the corresponding author upon reasonable request.
