# Supplementary material for: Heterodinuclear Mg(II)M(II) (M=Cr, Mn, Fe, Co, Ni, Cu and Zn) Complexes for the Ring Opening Copolymerization of Carbon Dioxide/Epoxide and Anhydride/Epoxide
Source: Chemistry. 2022 Feb 17;28(14):e202104198. doi: 10.1002/chem.202104198 (PMC9306976; doi:10.1002/chem.202104198)
Supplement: Supplementary file 1 — Supporting Information [file CHEM-28-0-s001.pdf]

Chemistry–A European Journal

Supporting Information

Heterodinuclear Mg(II)M(II) (M = Cr, Mn, Fe, Co, Ni, Cu and Zn) Complexes for the Ring Opening Copolymerization of Carbon Dioxide/Epoxide and Anhydride/Epoxide

Natalia V. Reis, Arron C. Deacy, Gloria Rosetto, Christopher B. Durr, and Charlotte K. Williams*

Figure S1: MALDI-ToF spectrum of complex **1**. 6
Figure S2: MALDI-ToF spectrum of complex **2**. 6
Figure S3: MALDI-ToF spectrum of complex **3**. 7
Figure S4: MALDI-ToF spectrum of complex **4**. 7
Figure S5: MALDI-ToF spectrum of complex **5**. 8
Figure S6: MALDI-ToF spectrum of complex **6**. 8
Figure S7: MALDI-ToF spectrum of complex **7**. 9
Figure S8: MALDI-ToF spectrum of complex **8**. 9
Figure S9: Cyclic Voltammagram of complex **1** vs. Fc^+/Fc (THF, 0.1 M [$^nBu_4N$][PF_6], 100 mV s^{-1}). 10
Figure S10: Cyclic Voltammagram of complex **2** vs. Fc^+/Fc (THF, 0.1 M [$^nBu_4N$][PF_6], 100 mV s^{-1}). 10
Figure S11: Cyclic Voltammagram of complex **3** vs. Fc^+/Fc (THF, 0.1 M [$^nBu_4N$][PF_6], 100 mV s^{-1}). 11
Figure S12: Cyclic Voltammagram of complex **4** vs. Fc^+/Fc (THF, 0.1 M [$^nBu_4N$][PF_6], 100 mV s^{-1}). 11
Figure S13: Cyclic Voltammagram of complex **5** vs. Fc^+/Fc (THF, 0.1 M [$^nBu_4N$][PF_6], 100 mV s^{-1}). 12
Figure S14: Cyclic Voltammagram of complex **6** vs. Fc^+/Fc (THF, 0.1 M [$^nBu_4N$][PF_6], 100 mV s^{-1}). 12
Figure S15: Cyclic Voltammagram of complex **7** vs. Fc^+/Fc (THF, 0.1 M [$^nBu_4N$][PF_6], 100 mV s^{-1}). 13
Figure S16: IR spectra of complexes **1-8**. 13
Figure S17: Illustration of literature 1 bar CO_2/cyclohexene oxide ROCOP catalysts as featured in Table 1. 14
Figure S18: Semi-logarithmic plot of [epoxide] versus time for the ROCOP of CO_2/CHO using **2**. 14
Figure S19: Semi-logarithmic plot of [epoxide] versus time for the ROCOP of CO_2/CHO using **3**. 15
Figure S20: Semi-logarithmic plot of [epoxide] versus time for the ROCOP of CO_2/CHO using **4**. 15
Figure S21: Semi-logarithmic plot of [epoxide] versus time for the ROCOP of CO_2/CHO using **5**. 16
Figure S22: Semi-logarithmic plot of [epoxide] versus time for the ROCOP of CO_2/CHO using **6**. 16
Figure S23: Semi-logarithmic plot of [epoxide] versus time for the ROCOP of CO_2/CHO using **7**. 17
Figure S24: Semi-logarithmic plot of [epoxide] versus time for the ROCOP of CO_2/CHO using **8**. 17
Figure S25: Representative example of a ^1H NMR spectrum for the ROCOP of CO_2/CHO using catalysts **1**-**8**. 18
Figure S26: Mass Spectrum (MALDI-ToF) of polycarbonate synthesised by catalyst **4** displaying two distributions; *α, ω*-hydroyl telechilic polyol (●) and Acetate-initiated polyol (■). 18
Figure S27: Cyclic Voltammagram of complex **Fe(II)Fe(III)** vs. Fc^+/Fc (THF, 0.1 M [$^nBu_4N$][PF_6], 100 mV s^{-1}). 19

Figure S28: ORTEP representation of the molecular structure of an **Fe(II)Fe(III)** complex obtained by single crystal X-ray diffraction experiments. Complex disorder issues and H-atoms (exception of NH) are omitted, for clarity, and the thermal ellipsoids are represented at 40 % probability. 20
Figure S29: Cyclic Voltammagram of complex **Fe(II)Fe(II)** vs. Fc^{+}/Fc (THF, 0.1 M [$^{n}Bu_4N$][PF_6], 100 mV s^{-1}). 22
Figure S30: MALDI-ToF of complex **Fe(II)Fe(II)**. 22
Figure S31: Illustration of literature NA/cyclohexene oxide ROCOP catalysts as featured in Table 2. 23
Figure S32: Plot of absorbance (1785 cm^{-1}) versus time for the ROCOP of NA/CHO using **2**. 23
Figure S33: Plot of absorbance (1785 cm^{-1}) versus time for the ROCOP of NA/CHO using **3**. 24
Figure S34: Plot of absorbance (1785 cm^{-1}) versus time for the ROCOP of NA/CHO using **4**. 24
Figure S35: Plot of absorbance (1785 cm^{-1}) versus time for the ROCOP of NA/CHO using **5**. 25
Figure S36: Plot of absorbance (1785 cm^{-1}) versus time for the ROCOP of NA/CHO using **6**. 25
Figure S37: Plot of absorbance (1785 cm^{-1}) versus time for the ROCOP of NA/CHO using **7**. 26
Figure S38: Plot of absorbance (1785 cm^{-1}) versus time for the ROCOP of NA/CHO using **8**. 26
Figure S39: Plot of first row transition metals bond dissociation energies (BDEs), electronegativities (Mulliken) and oxophilicity. 27
Figure S40: Plots of water exchange rate constants (LHS) and hydrolysis constants (RHS) against metal ion and their observed activity in copolymerisation of CO2/CHO. 27

Experimental

General Comments:

All solvents and reagents were obtained from commercial sources and used as received unless states otherwise. All metal complexes were synthesised under anhydrous conditions, using a nitrogen-vacuum Schlenk line or a nitrogen-filled glovebox. Common organic solvents used in complex synthesis were obtained from a SPS system, degassed by three freeze-pump-thaw cycles and stored over activated 3 Å or 4 Å molecular sieves under nitrogen. Cyclohexene oxide was dried over calcium hydride for 5 days and purifies via fractional distillation under inert atmosphere, it was then degassed by three freeze-pump-thaw cycles and stored under nitrogen. NA was purified via solvent extraction into benzene, recrystalisation in chloroform followed by sublimation. All anhydrous deuterated solvents were dried over calcium hydride overnight, and purified via vacuum transfer and stored under nitrogen with 4 molecular sieves. All chemicals are purchased from Merck unless stated otherwise. NMR experiments were performed on a Bruker Avance III HD nanobay NMR with 9.4 T magnet, 400 MHz. Research grade carbon dioxide (BOC, 99.99 %) was dried through two drying columns (Micro Torr, Model number: MC1-804FV) in series. The norbornene- and phthalic-anhydride was purified by solvent extraction into benzene, recrystallized in chloroform followed by three sublimations. In situ ATR-IR measurements were performed on a Mettler-Toledo ReactIR ic.10 spectrometer equipped with a MCT detector and a silver halide DiComp probe. The IR measurements were performed in a Varian FTS-7000 Fourier Transform Infrared Spectrometer. MALDI-ToF analysis were performed on a Bruker MALDI Autroflex Speed and the data was analysed using mmass software. Matrixes used were trans-2-[3-(4-tert-butylphenyl)-2-methyl-2-propenylidene]-malonitrile (complexes) and dithranol (polymer). Size exclusion chromatrography was carried out on a two mixed bed PSS SDV linear S column in series, with THF as the eluent at a flow rate of 1mL/min on a Shimadzu LC-20AD instrument at 40 °C. Molar masses were determined by comparison against polystyrene standards. Elemental analysis was carried out by Mr Stephen Boyer at the London Metropolitan University. Cyclic voltammograms were recorded in a nitrogen glovebox using a PalmSens EmStat Blue potentiostat under computer control with three electrodes: Au disc (2.0 mm2) as the working electrode, Pt wire as the counter electrode and a Ag wire as the pseudo-reference electrode. The measurements were performed with a solution of 5 mM complex with 0.1 M $[^{n}Bu_4N][PF_6]$ in 10mL of THF. Potentials are referenced to the Fc^+/Fc redox couple.

The general procedure for X-ray diffraction experiments in our lab has been described previously. [1,2] Crystals of **Fe(II)Fe(III)** were isolated and coated in a pool of Fomblin®. A single crystal mounted on a MiTeGen Micromount™ and centered in the X-ray beam and the sample was cooled with an Oxford Cryosystems Cryostream to a temperature of 150 K prior to data collection. Data was obtained with Cu κ_α (λ = 1.5417 Å) radiation on an Oxford Diffraction Supernova Diffractometer. Data reduction was conducted with CrysAlisPRO,[3] followed by structural solution with SHELXT[4] and subsequent refinement with SHELXL.[5] WinGX was used to compile the final crystallographic information file (CIF) and as the graphical user interface.[6] Tables were generated from the CIF using online tools from the IUCr.[7]

The structure **Fe(II)Fe(III)** was registered with the Cambridge Structural Database. CCDC: 2108011 contains the supplementary crystallographic data for this paper. The data can be obtained free of charge from The Cambridge Crystallographic Data Centre via www.ccdc.cam.ac.uk/structures.

General Catalyst Synthesis:

$Mg(N(Si(CH_3)_3)_2)_2$ (0.31 g, 0.91 mmol) was added to H_2L (0.5 g, 0.91 mmol) in THF (15 mL) and stirred for 2 h. $M(OAc)_2$ (0.91 mmol) was added to the reaction solution and stirred for 16 hours at 100 °C in a J-Young ampoule. The solution was reduced in *vacuo* and the product was washed with pentane (3 x 20 mL) to afford the final complex.

Catalyst **1**: White powder (0.46 g, 0.64 mmol, 70 %) Elemental analysis for $C_{38}H_{60}Mg_2N_4O_6$ (716.42 g mol^{-1}): Calculated; C, 63.6; H, 8.6; N, 7.6 %. Found; C, 63.1; H, 8.7; N, 7.7 %. MS (MALDI-ToF): 657.27 m/z $[LMgMg(OAc)]^+$. IR (cm^{-1}): 3308 (v_{NH}), 1595 ($v_{COO\text{-}as}$) 1423 ($v_{COO\text{-}sym}$) and 924 (v_{COO})

Catalyst **2**: $Cr(OAc)_2$ was added at -78 °C. Dark blue powder (0.34g, 0.46 mmol, 50 %). MS (MALDI-ToF): 685.19 m/z $[LMgCr(OAc)]^+$. IR (cm^{-1}): 3307 (v_{NH}), 1593 ($v_{COO\text{-}as}$) 1421 ($v_{COO\text{-}sym}$) and 924 (v_{COO})

Catalyst **3**: Yellow powder (0.46 g, 0.62 mmol, 68 %) Elemental analysis for $C_{38}H_{60}MgMnN_4O_6$ (747.37 g mol^{-1}): Calculated; C, 61.0; H, 8.0; N, 7.5 %. Found; C, 60.9; H, 8.0; N, 7.5 %. MS (MALDI-ToF): 688.19 m/z $[LMgMn(OAc)]^+$. IR (cm^{-1}): 3308 (v_{NH}), 1583 ($v_{COO\text{-}as}$) 1415 ($v_{COO\text{-}sym}$) and 926 (v_{COO})

Catalyst **4**: Brown powder (0.51 g, 0.68 mmol, 75 %) Elemental analysis for $C_{38}H_{60}MgFeN_4O_6$ (748.37 g mol^{-1}): Calculated; C, 60.9; H, 8.0; N, 7.5 %. Found; C, 61.5; H, 8.7; N, 7.7 %. MS (MALDI-ToF): 689.28 m/z $[LMgFe(OAc)]^+$. IR (cm^{-1}): 3308 (v_{NH}), 1587 ($v_{COO\text{-}as}$) 1421 ($v_{COO\text{-}sym}$) and 924 (v_{COO})

Catalyst **5**: Pink powder (0.52 g, 0.68 mmol, 75 %) Elemental analysis for $C_{38}H_{60}MgCoN_4O_6$ (751.37 g mol^{-1}): Calculated; C, 60.7; H, 8.0; N, 7.5 %. Found; C, 60.5; H, 7.9; N, 7.3 %. MS (MALDI-ToF): 692.26 m/z $[LMgCo(OAc)]^+$. IR (cm^{-1}): 3300 (v_{NH}), 1587 ($v_{COO\text{-}as}$) 1419 ($v_{COO\text{-}sym}$) and 925 (v_{COO})

Catalyst **6**: Light blue powder (0.53 g, 0.71 mmol, 78 %) Elemental analysis for $C_{38}H_{60}MgNiN_4O_6$ (750.37 g mol^{-1}): Calculated; C, 60.7; H, 8.1; N, 7.5 %. Found; C, 60.3; H, 8.1; N, 7.3 %. MS (MALDI-ToF): 691.20 m/z $[LMgNi(OAc)]^+$. IR (cm^{-1}): 3290 (v_{NH}), 1587 ($v_{COO\text{-}as}$) 1419 ($v_{COO\text{-}sym}$) and 937 (v_{COO})

Catalyst **7**: Green powder (0.45 g, 0.59 mmol, 65 %) Elemental analysis for $C_{38}H_{60}MgCuN_4O_6$ (755.37 g mol^{-1}): Calculated; C, 60.3; H, 8.0; N, 7.4 %. Found; C, 60.3; H, 8.0; N, 7.4 %. MS (MALDI-ToF): 696.28 m/z $[LMgCu(OAc)]^+$. IR (cm^{-1}): 3306 (v_{NH}), 1591 ($v_{COO\text{-}as}$) 1421 ($v_{COO\text{-}sym}$) and 914 (v_{COO})

Catalyst **8**: White powder (0.30 g, 0.42 mmol, 50 %) Elemental analysis for $C_{38}H_{60}MgN_4O_6Zn$ (756.37 g mol^{-1}): Calculated; C, 60.2; H, 8.0; N, 7.4 %. Found; C, 59.4; H, 7.6; N, 7.0 %. MS (MALDI-ToF): 697.18 m/z $[LMgZn(OAc)]^+$. IR (cm^{-1}): 3300 (v_{NH}), 1591 ($v_{COO\text{-}as}$) 1419 ($v_{COO\text{-}sym}$) and 922 (v_{COO})

Catalyst **Fe(II)Fe(III):** $Fe(OAc)_2$ (0.19 g, 1.08 mmol) was added to H_2L (0.30 g, 0.54 mmol) in THF (10 mL) and stirred for 16 hours at 100 °C. The volatiles were removed and the product washed with toluene (3 x 10 mL) and then with pentane (3 x 10 mL) and dried overnight to yield a purple powder (0.27 g, 0.32 mmol, 60 %). Elemental analysis for $C_{40}H_{63}Fe_2N_4O_8$ (839.33 g mol^{-1}): Calculated; C, 57.2; H, 7.6; N, 6.7 %. Found; C, 57.4; H, 7.6; N, 7.0 %. MS

(MALDI-ToF): 721.31 m/z $[LFeFe(OAc)]^+$. IR (cm^{-1}): 3296 (ν_{NH}), 1596 ($\nu_{COO\text{-}as}$) 1425 ($\nu_{COO\text{-}sym}$) and 925 (ν_{COO})

Catalyst **Fe(II)Fe(II):** $K(N(Si(CH_3)_3)_2)$ (0.22 g, 1.08 mmol) was added to H_2L (0.30 g, 0.54 mmol) in THF (10 mL) and stirred for 2 hours. Volatiles were removed and the LK_2 product washed with pentane. $Fe(OAc)_2$ (0.19 g, 1.08 mmol) was added to LK_2 in THF (10 mL) and stirred at 100 °C for 16 hours. The solution was filtered (removing KOAc salts) and volatiles were removed in *vacuo*. The product was washed with pentane (3 x 10 mL) and dried to yield a red solid (0.31 g, 0.42 mmol, 40 %). MS (MALDI-ToF): 721.31 m/z $[LFeFe(OAc)]^+$. IR (cm^{-1}): 3298 (ν_{NH}), 1595 ($\nu_{COO\text{-}as}$) 1421 ($\nu_{COO\text{-}sym}$) and 921 (ν_{COO})

General CO_2/CHO Copolymerization Procedure:

A Schlenk was charged with 6.0 mL of CHO (60 mmol), catalyst (0.015 mmol) and 1,2-cyclohexane diol (0.03 mmol) in a glovebox. The Schlenk was connected to a CO_2-N_2-Vacuum triple manifold Schlenk line through several vacuum-CO_2 cycles. The reaction was placed under 1 bar CO_2 atmosphere, ATR-IR probe inserted and heated to the reaction temperature (100 °C). After completion, the reaction was quenched through cooling to 25 °C and opening to air. The conversion was obtained by ^1H NMR spectroscopy of an aliquot taken at the end of the reaction. The polymer was purified by precipitation into acidified methanol (1M benzoic acid) and dried under vacuum.

General Anhydride/CHO Copolymerization Procedure:

A Schlenk was charged with 3.0 mL of CHO (30 mmol), catalyst (0.015 mmol) and anhydride (1.5 mmol) in a glovebox. The Schlenk was connected to a CO_2-N_2-Vacuum triple manifold Schlenk line through several vacuum-N_2 cycles. The reaction was placed under 1 bar N_2 atmosphere, ATR-IR probe inserted and heated to the reaction temperature (100 °C). After completion, the reaction was quenched through cooling to 25 °C and opening to air. The conversion was obtained by ^1H NMR spectroscopy of an aliquot taken at the end of the reaction. The polymer was purified by precipitation into acidified methanol (1M benzoic acid) and dried under vacuum.

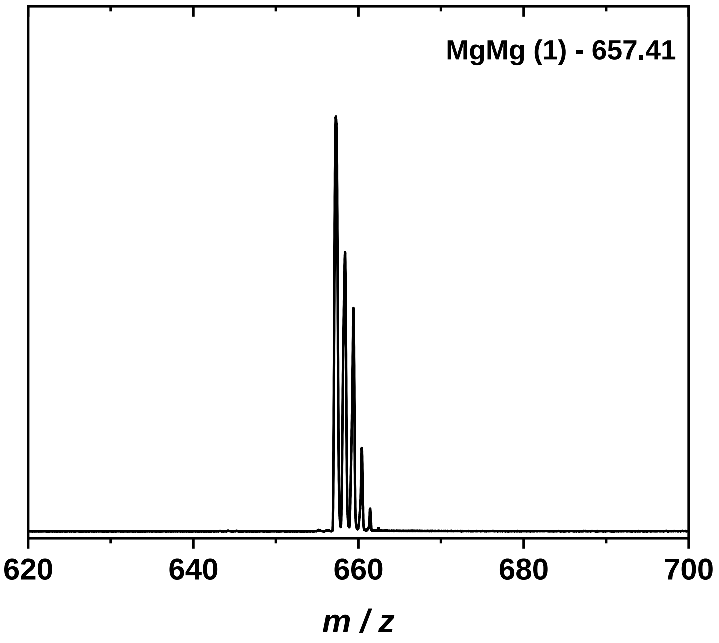

Figure S1: MALDI-ToF spectrum of complex **1**.

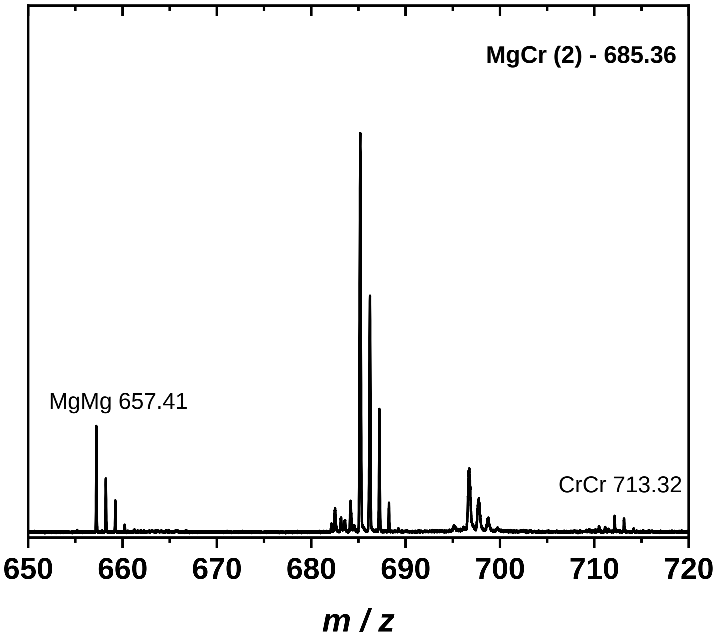

Figure S2: MALDI-ToF spectrum of complex **2**.

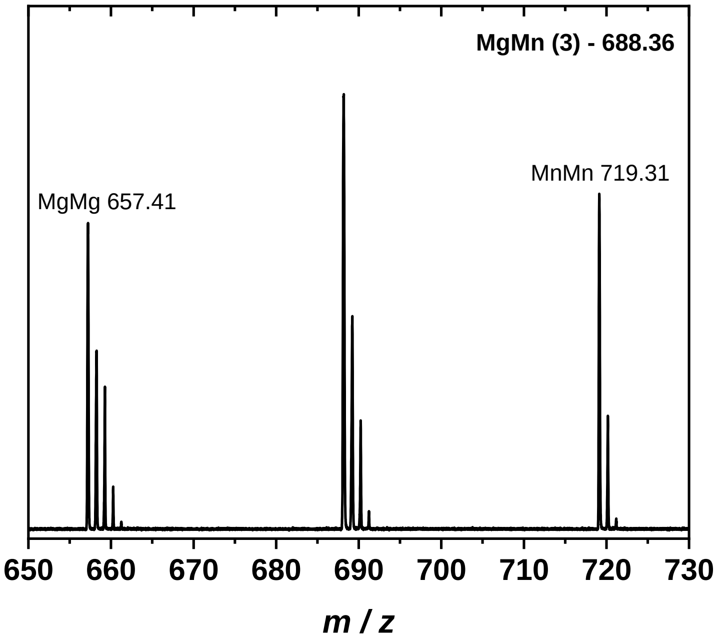

Figure S3: MALDI-ToF spectrum of complex **3**.

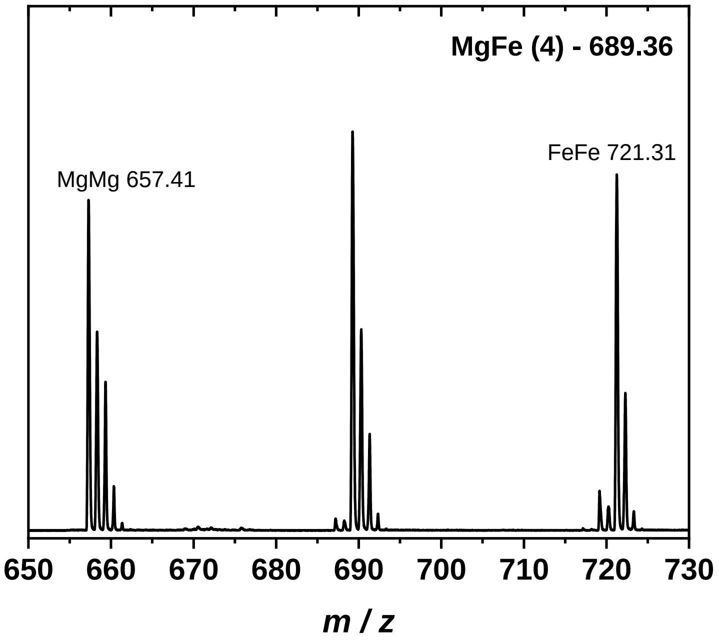

Figure S4: MALDI-ToF spectrum of complex **4**.

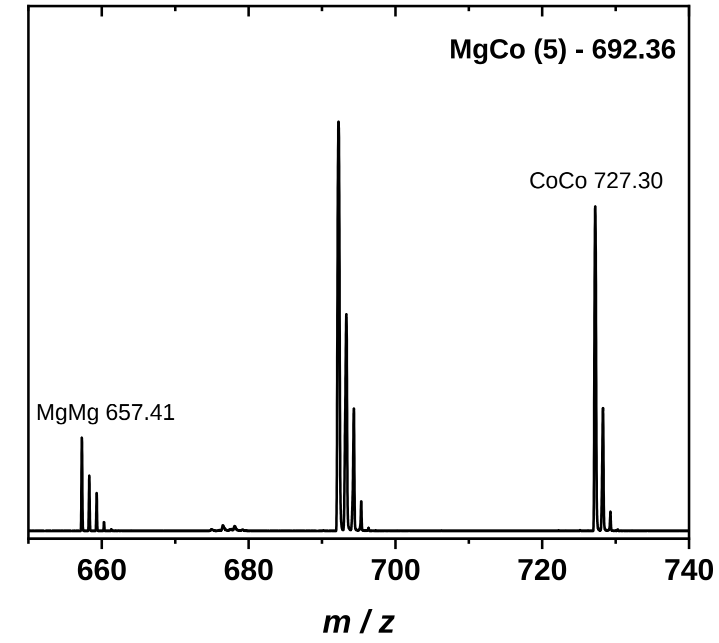

Figure S5: MALDI-ToF spectrum of complex **5**.

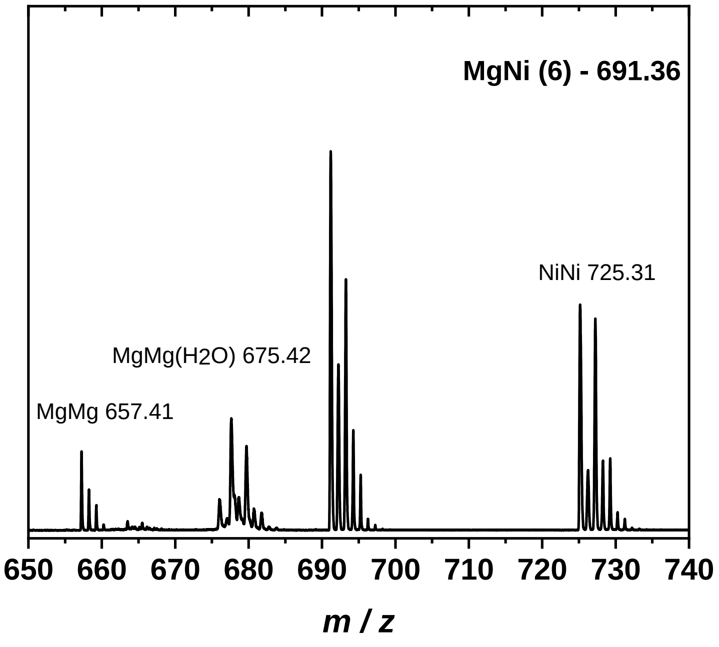

Figure S6: MALDI-ToF spectrum of complex **6**.

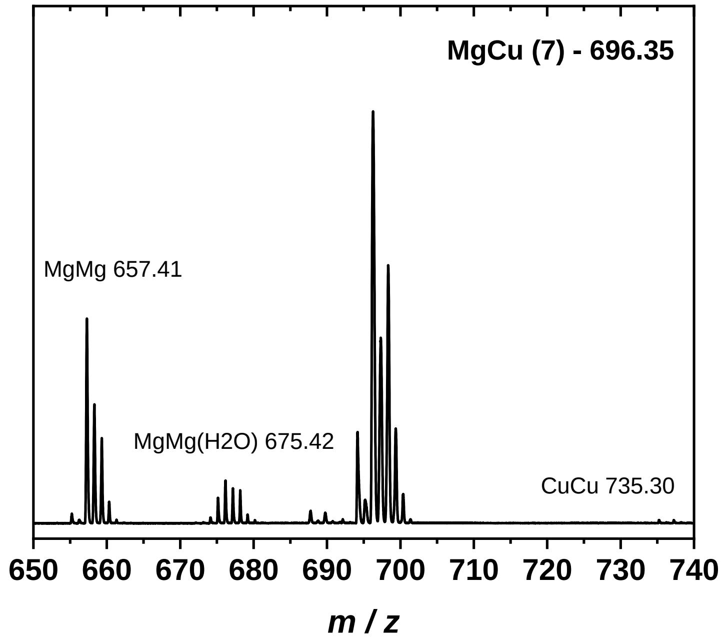

Figure S7: MALDI-ToF spectrum of complex **7**.

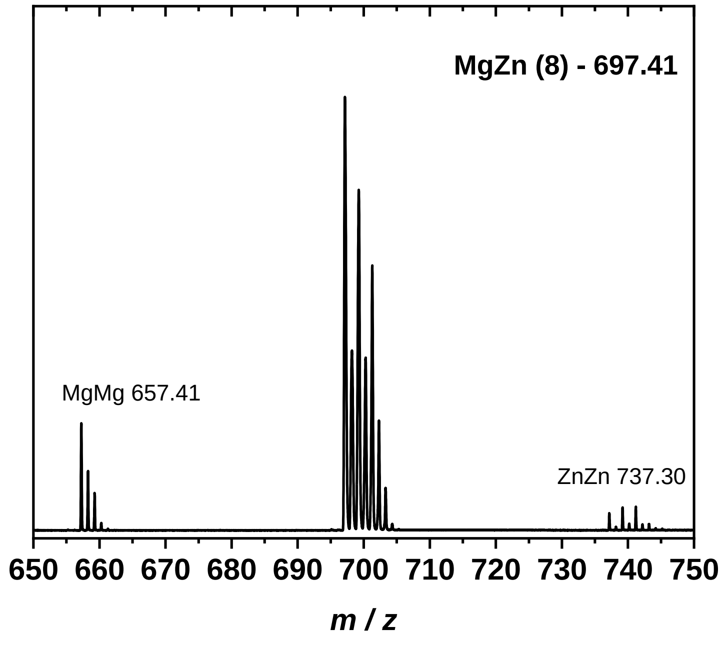

Figure S8: MALDI-ToF spectrum of complex **8**.

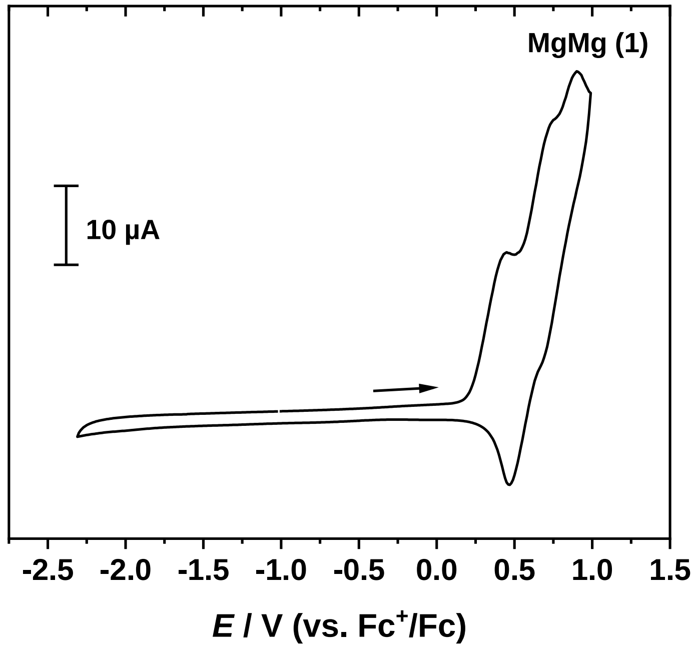

Figure S9: Cyclic Voltammagram of complex **1** vs. Fc^+/Fc (THF, 0.1 M [^{n}Bu_4N][PF_6], 100 mV s^{-1}).

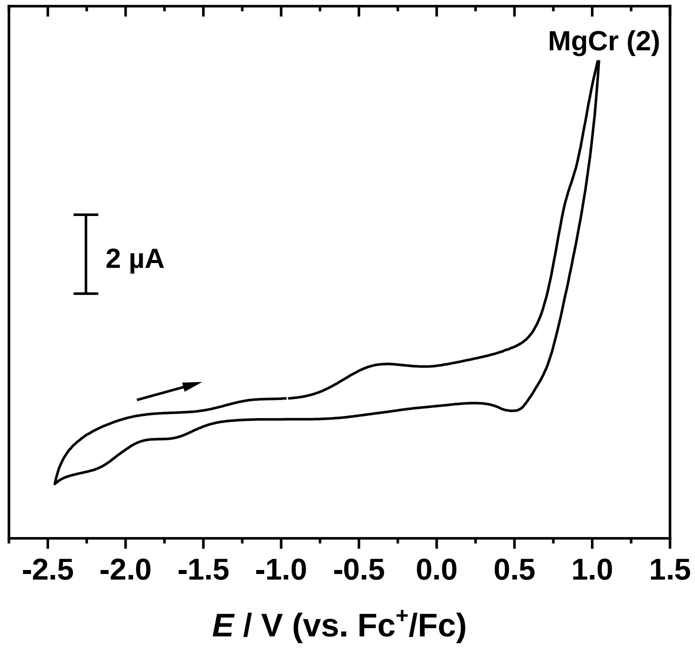

Figure S10: Cyclic Voltammagram of complex **2** vs. Fc^+/Fc (THF, 0.1 M [^{n}Bu_4N][PF_6], 100 mV s^{-1}).

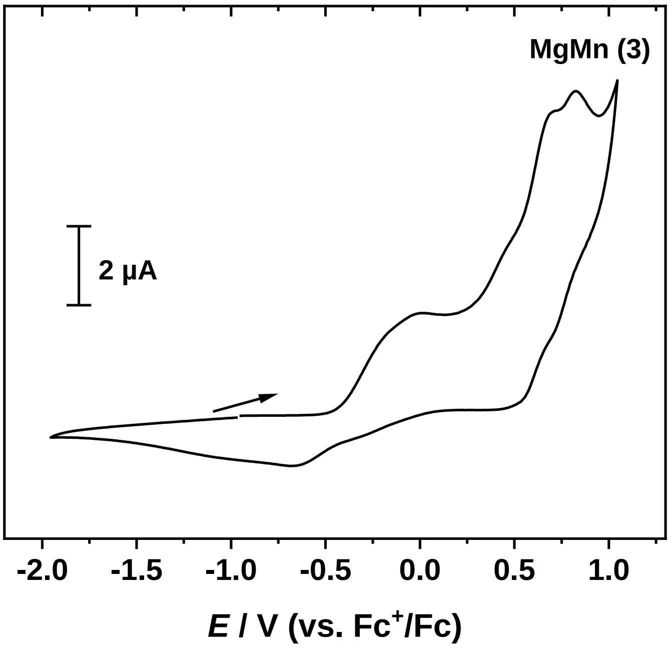

Figure S11: Cyclic Voltammagram of complex **3** vs. Fc^+/Fc (THF, 0.1 M [$^nBu_4N$][PF_6], 100 mV s^{-1}).

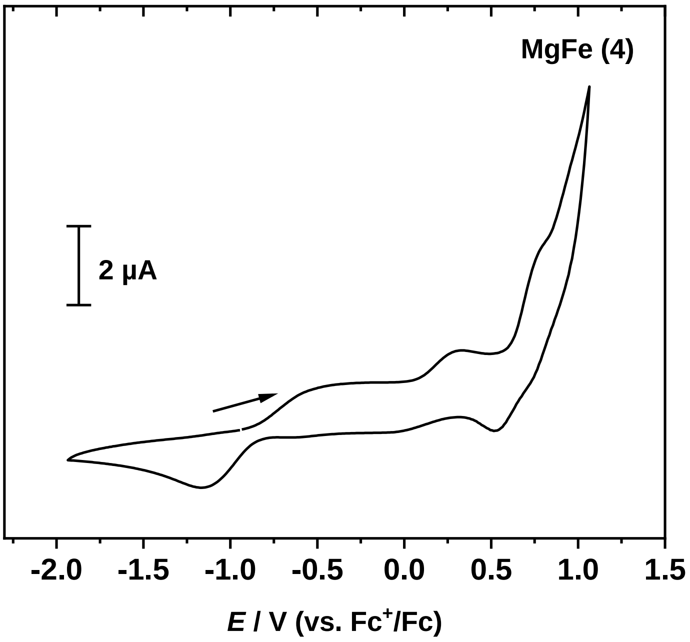

Figure S12: Cyclic Voltammagram of complex **4** vs. Fc^+/Fc (THF, 0.1 M [$^nBu_4N$][PF_6], 100 mV s^{-1}).

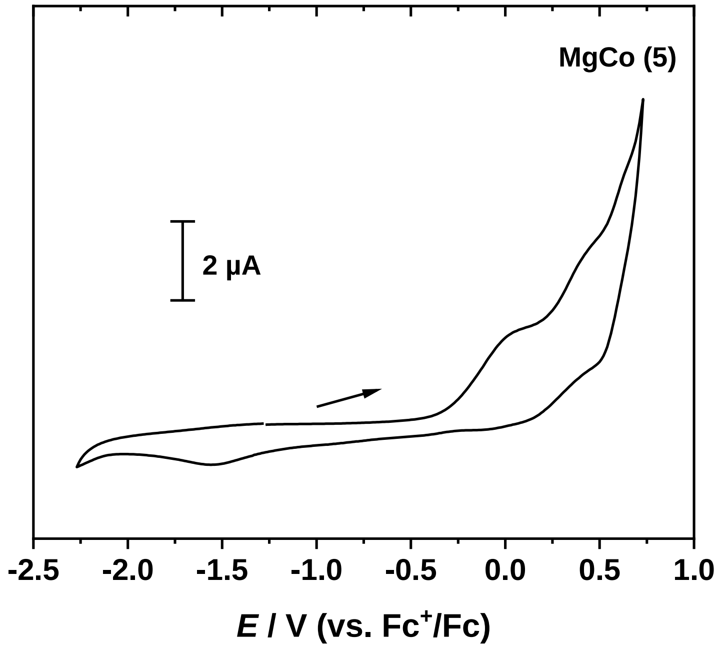

Figure S13: Cyclic Voltammagram of complex **5** vs. Fc^{+}/Fc (THF, 0.1 M [$^{n}Bu_4N$][PF_6], 100 mV s^{-1}).

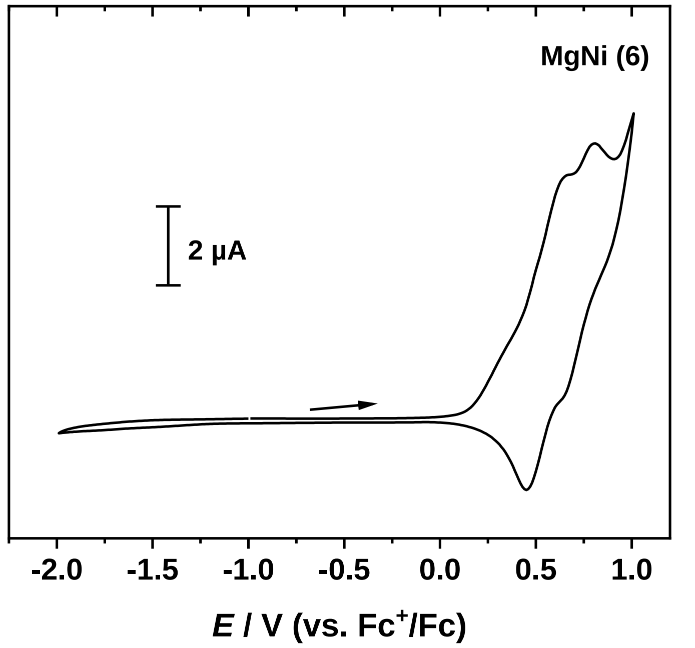

Figure S14: Cyclic Voltammagram of complex **6** vs. Fc^{+}/Fc (THF, 0.1 M [$^{n}Bu_4N$][PF_6], 100 mV s^{-1}).

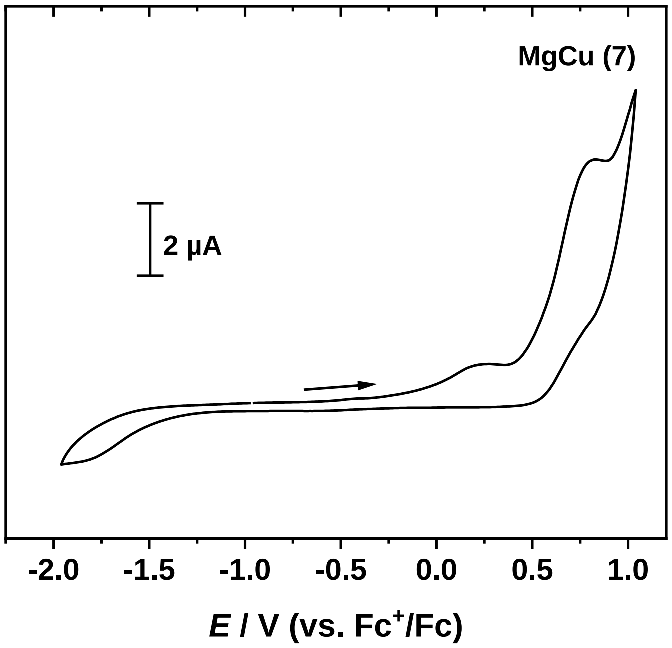

Figure S15: Cyclic Voltammagram of complex **7** vs. Fc^{+}/Fc (THF, 0.1 M [$^{n}Bu_4N$][PF_6], 100 mV s^{-1}).

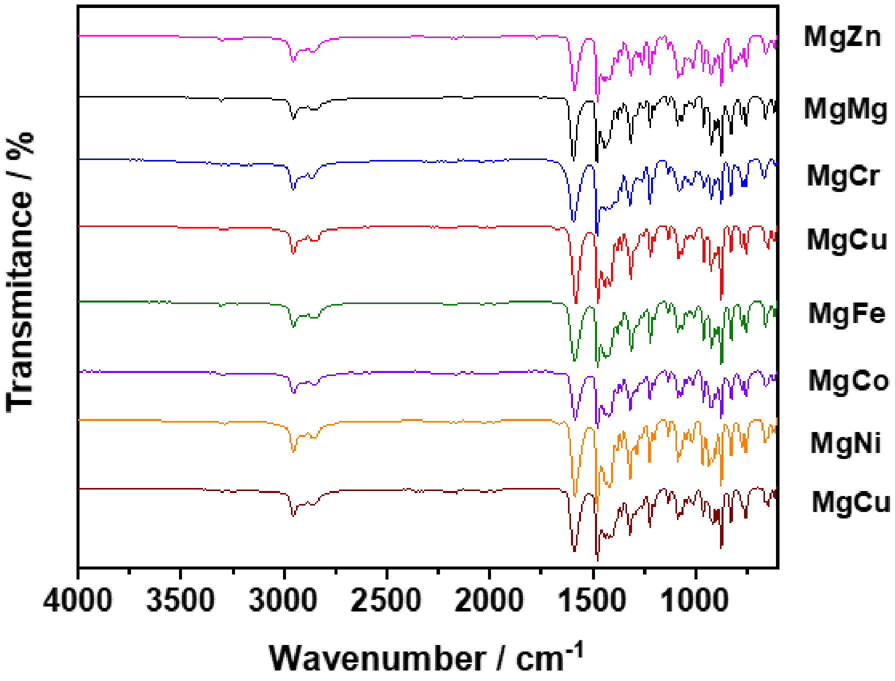

Figure S16: IR spectra of complexes **1-8**.

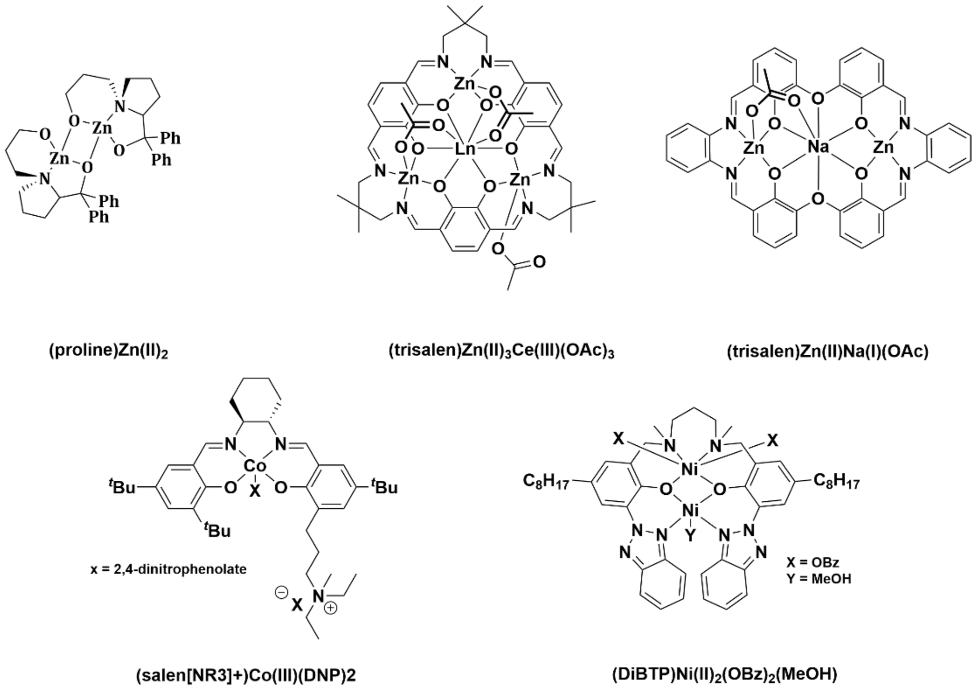

Figure S17: Illustration of literature 1 bar CO_2/cyclohexene oxide ROCOP catalysts as featured in Table 1.

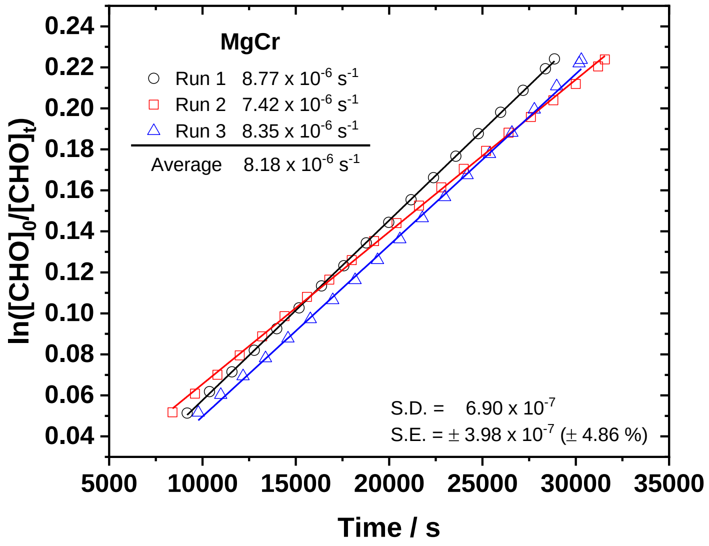

Figure S18: Semi-logarithmic plot of [epoxide] versus time for the ROCOP of CO_2/CHO using **2**.

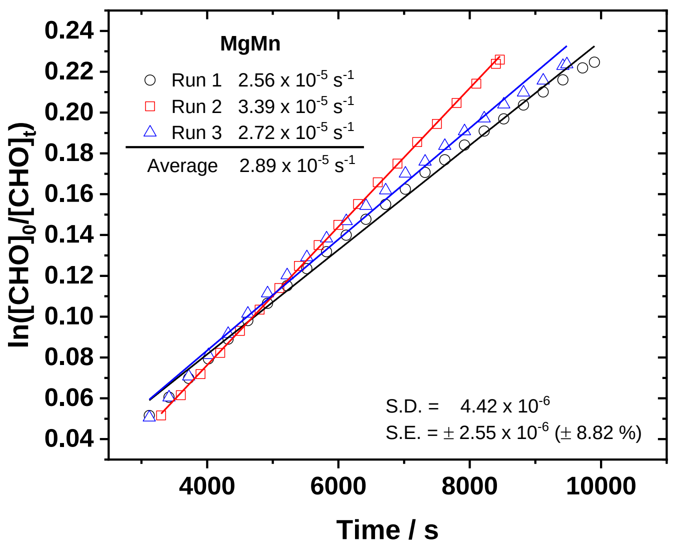

Figure S19: Semi-logarithmic plot of [epoxide] versus time for the ROCOP of CO_2/CHO using **3**.

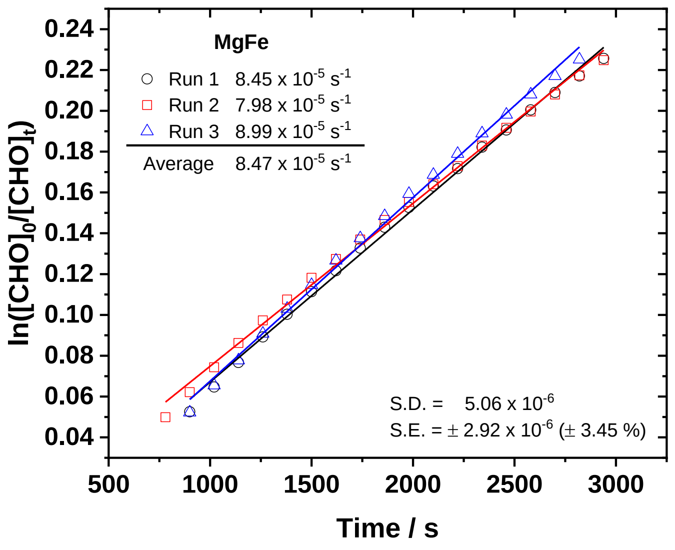

Figure S20: Semi-logarithmic plot of [epoxide] versus time for the ROCOP of CO_2/CHO using **4**.

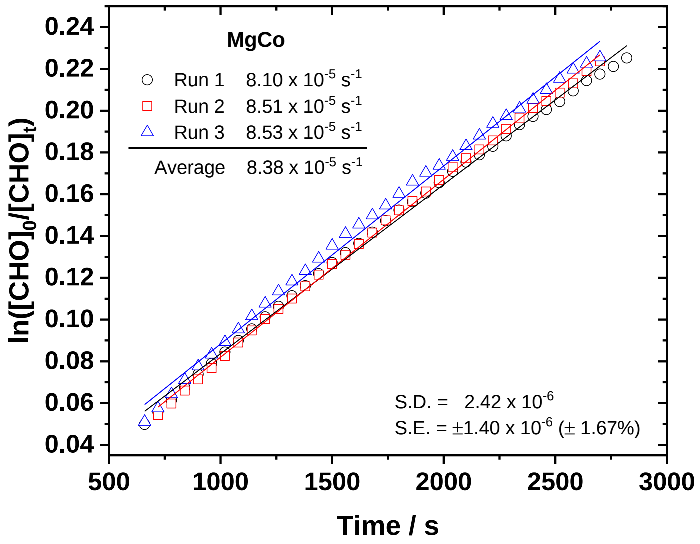

Figure S21: Semi-logarithmic plot of [epoxide] versus time for the ROCOP of CO_2/CHO using **5**.

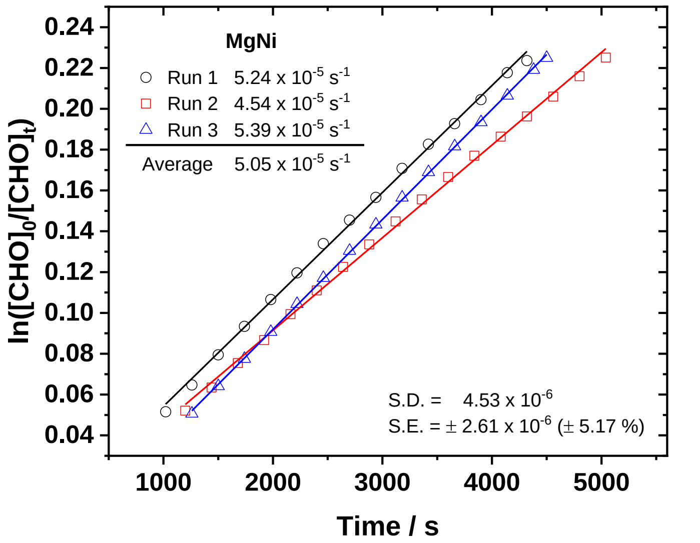

Figure S22: Semi-logarithmic plot of [epoxide] versus time for the ROCOP of CO_2/CHO using **6**.

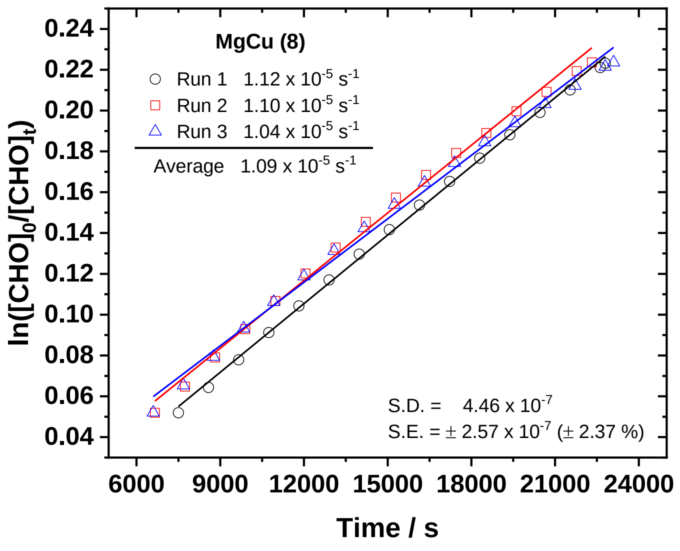

Figure S23: Semi-logarithmic plot of [epoxide] versus time for the ROCOP of CO_2/CHO using **7**.

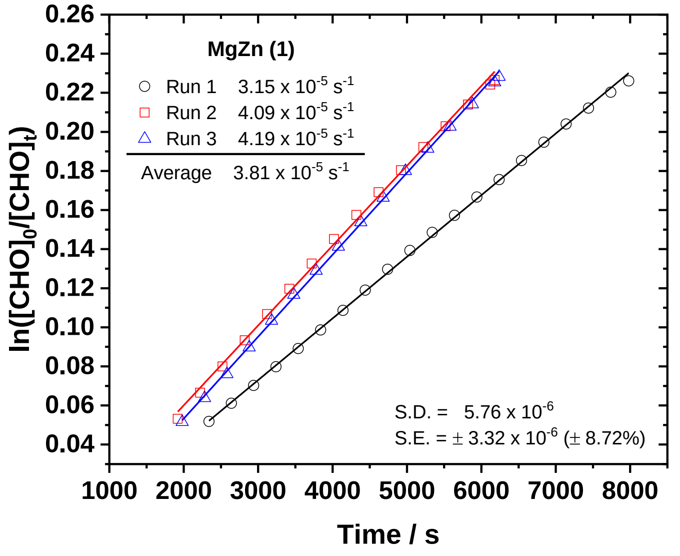

Figure S24: Semi-logarithmic plot of [epoxide] versus time for the ROCOP of CO_2/CHO using **8**.

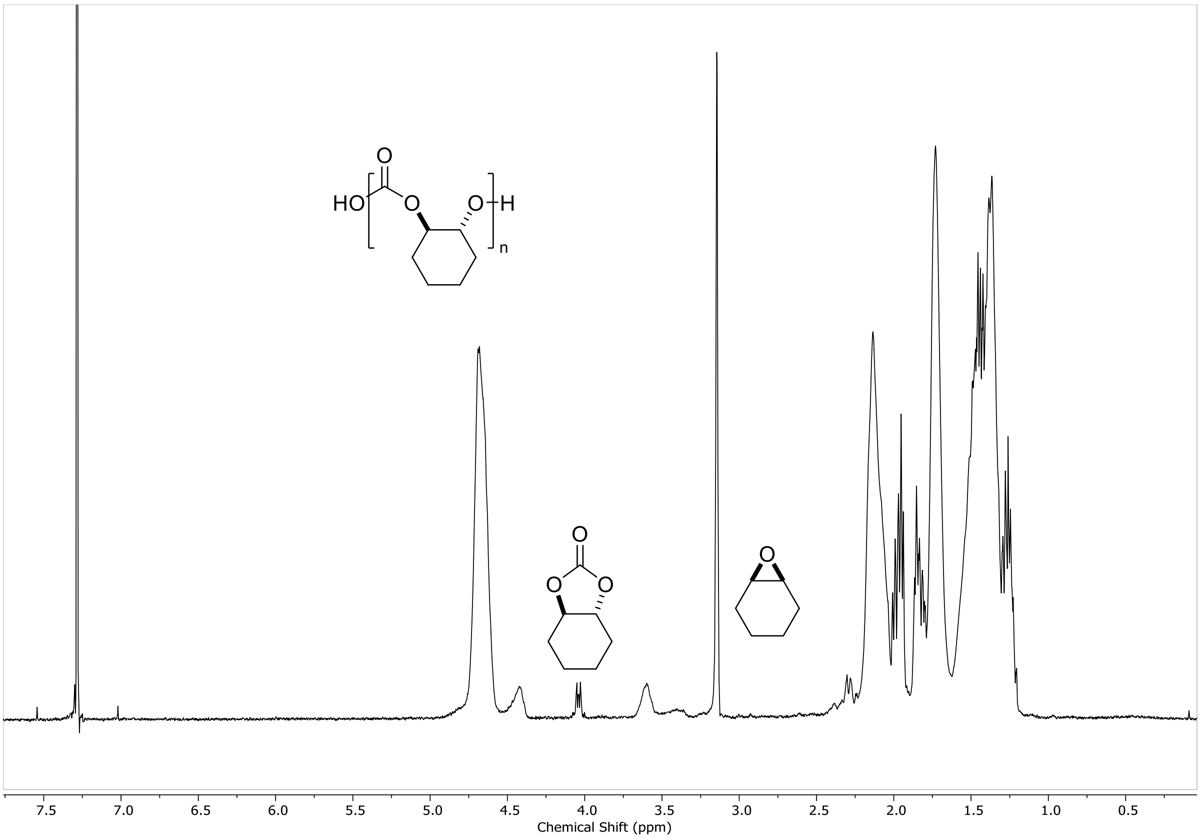

Figure S25: Representative example of a ^{1}H NMR spectrum for the ROCOP of CO_2/CHO using catalysts **1**-**8**.

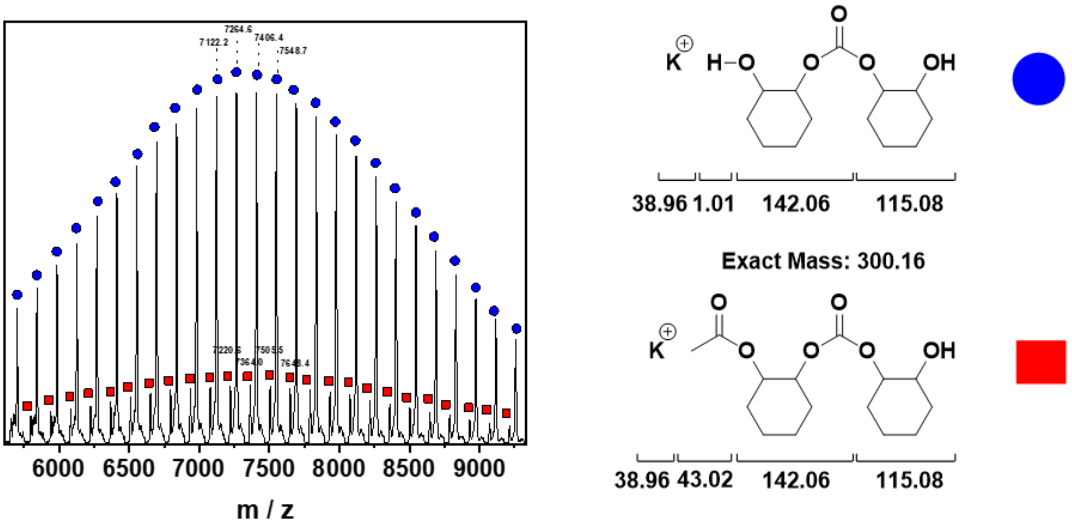

Figure S26: Mass Spectrum (MALDI-ToF) of polycarbonate synthesised by catalyst **4** displaying two distributions; *α, ω*-hydroyl telechilic polyol (●) and Acetate-initiated polyol (■).

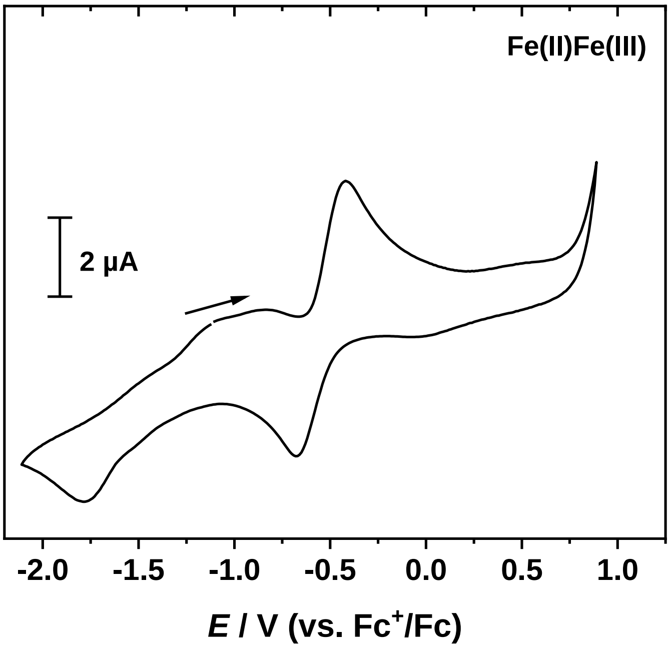

Figure S27: Cyclic Voltammagram of complex **Fe(II)Fe(III)** vs. Fc^+/Fc (THF, 0.1 M [$^nBu_4N$][PF_6], 100 mV s^{-1}).

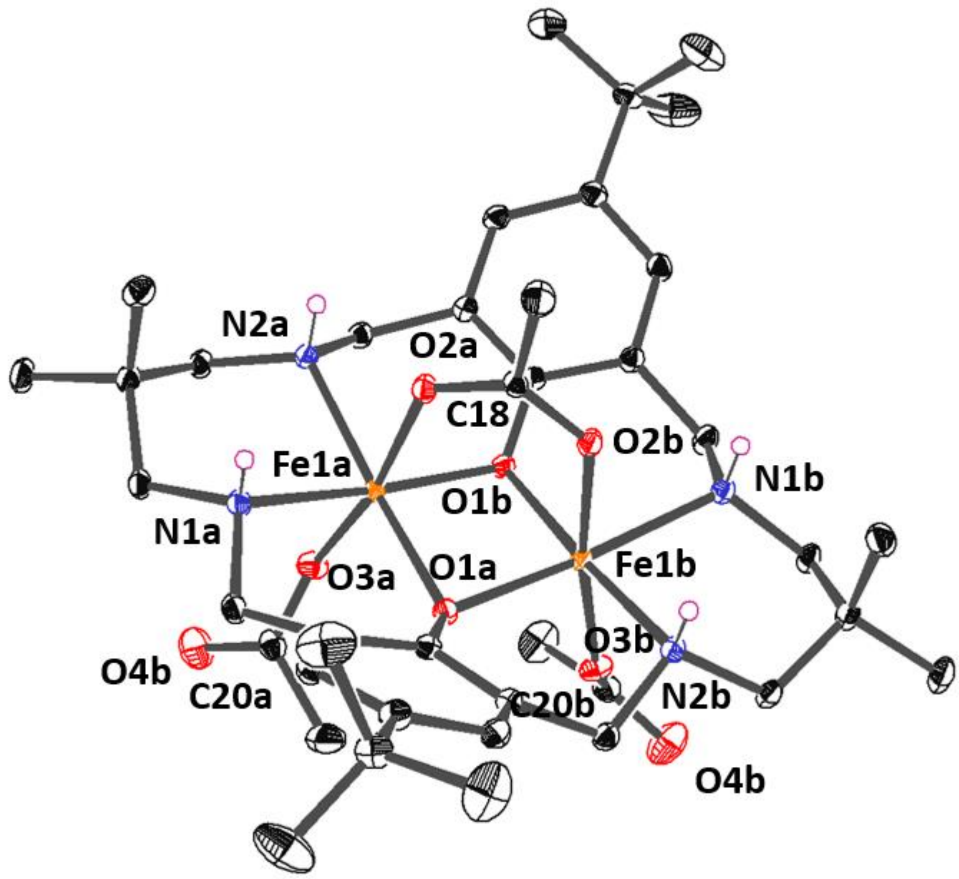

Figure S28: ORTEP representation of the molecular structure of an **Fe(II)Fe(III)** complex obtained by single crystal X-ray diffraction experiments. Complex disorder issues and H-atoms (exception of NH) are omitted, for clarity, and the thermal ellipsoids are represented at 40 % probability.

Table S1: Selected bond lengths (Å) and angles (°) for the **Fe(II)Fe(III)** complex.

**Bond**	**Bond Length (Å)**	**Bond**	**Bond Angle (°)**
N (1a) – Fe (1a)	2.1964(15)	N (1a) – Fe (1a) – O (1a)	173.04(6)
N (2a) – Fe (1a)	2.1912(15)	N (2a) – Fe (1a) – O (1b)	174.12(5)
O (1a) – Fe (1a)	2.0602(13)	O (2a) – Fe (1a) – O (3a)	170.79(6)
O (1b) – Fe (1a)	2.0640(13)	O (2a) – C (18) – O (2b)	125.3(2)
O (2a) – Fe (1a)	2.0946(13)	O (3a) – C (20) – O (4a)	123.1(2)
O (3a) – Fe (1a)	1.9644(13)		
O (2a) – C (18)	1.2593(17)		
O (2b) – C (18)	1.2593(17)	**Metal – Metal**	**Distance (Å)**
O (3a) – C (20)	1.275(2)		
O (4a) – C (20)	1.226(3)	Fe (1a) – Fe (1b)	2.8999(5)

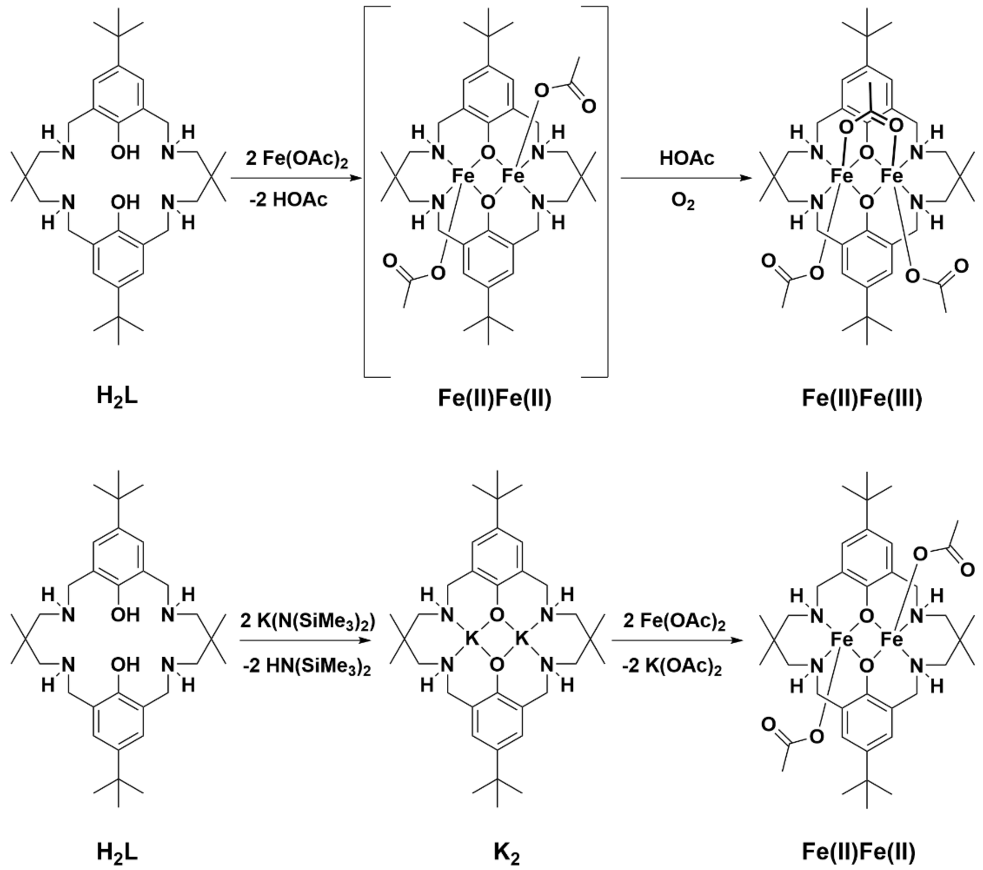

Scheme S1: Illustration of the synthesis of both Fe(II)Fe(III) and Fe(II)Fe(II) complexes.

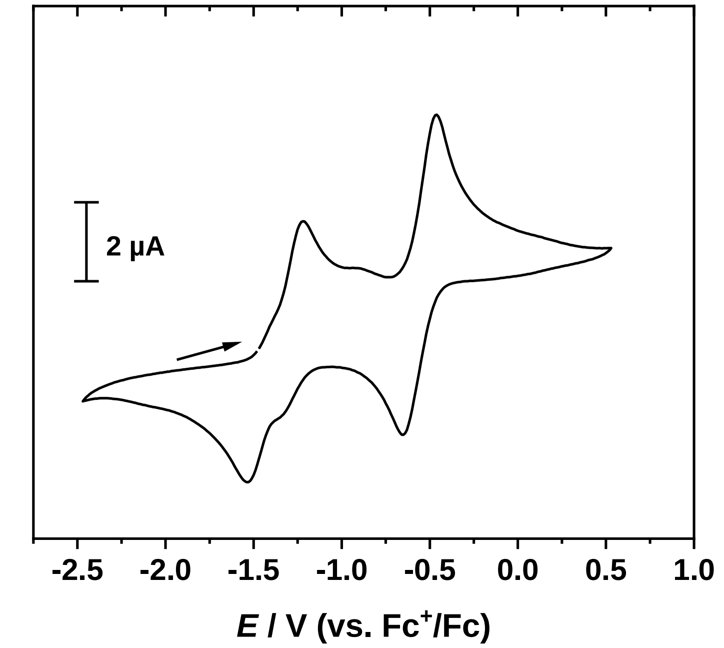

Figure S29: Cyclic Voltammagram of complex **Fe(II)Fe(II)** vs. Fc^{+}/Fc (THF, 0.1 M [$^{n}Bu_4N$][PF_6], 100 mV s^{-1}).

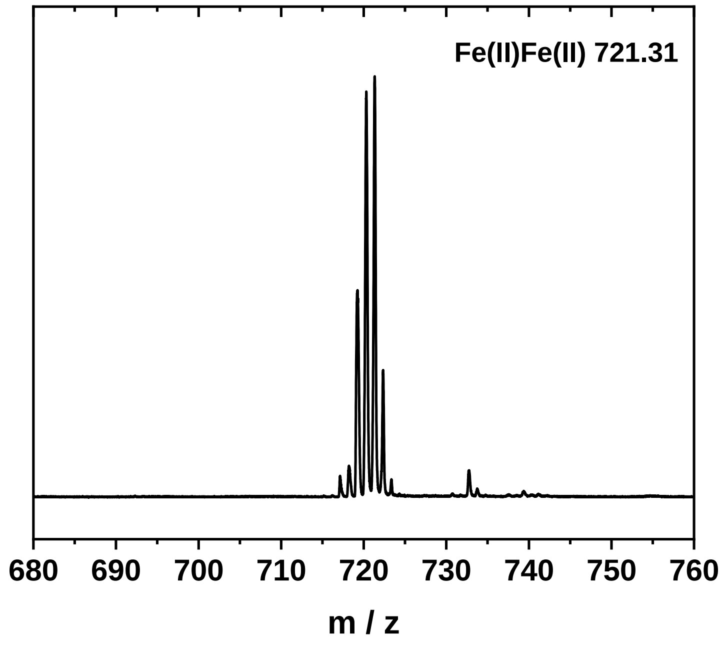

Figure S30: MALDI-ToF of complex **Fe(II)Fe(II)**.

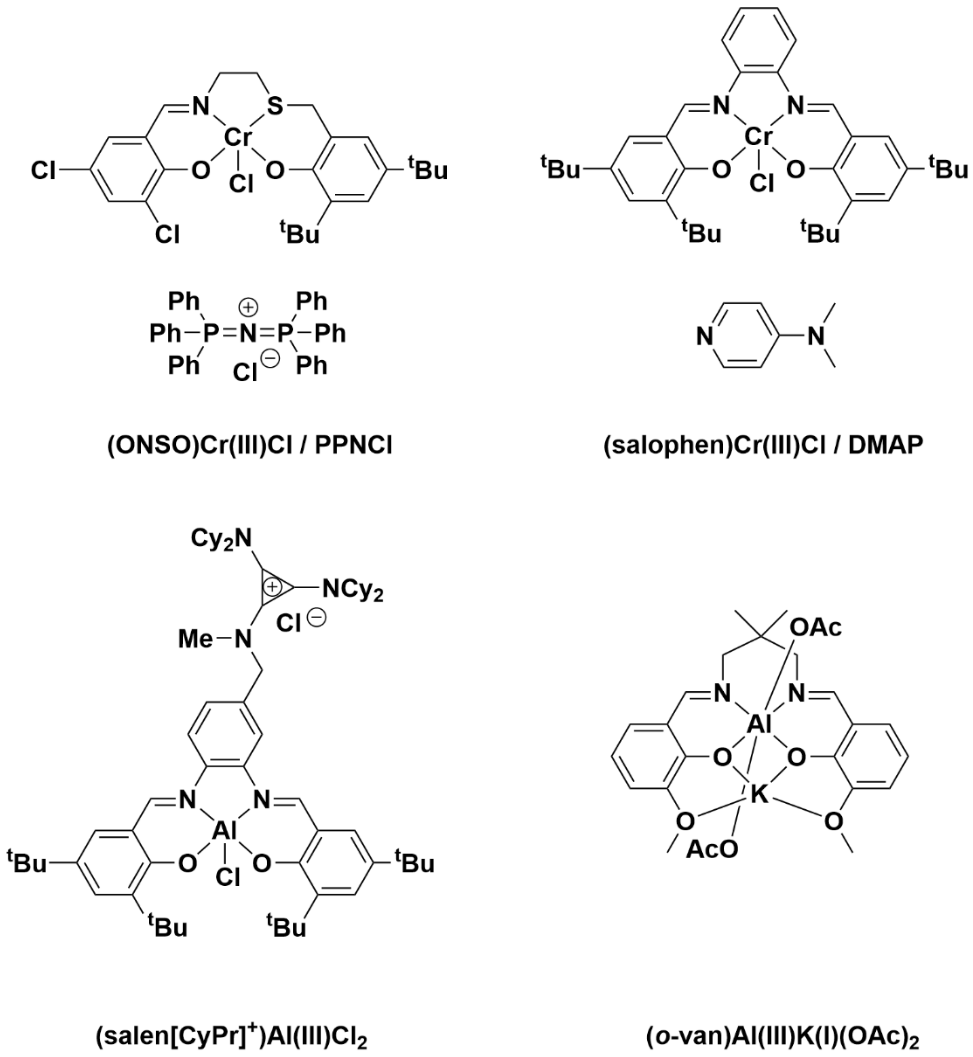

Figure S31: Illustration of literature NA/cyclohexene oxide ROCOP catalysts as featured in Table 2.

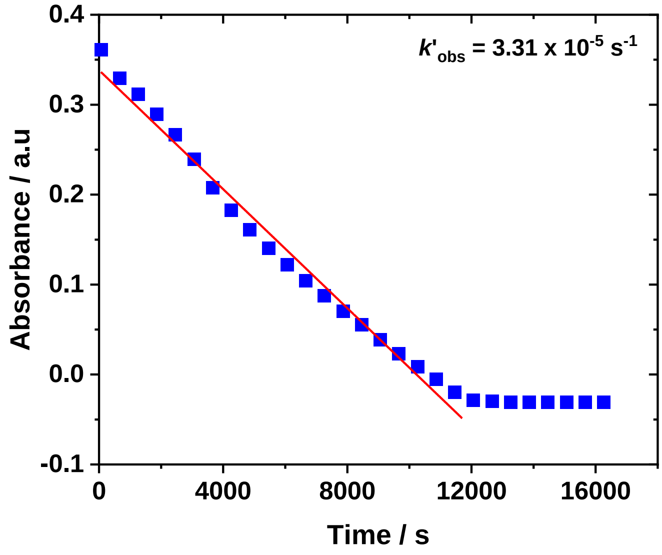

Figure S32: Plot of absorbance (1785 cm^{-1}) versus time for the ROCOP of NA/CHO using **2**.

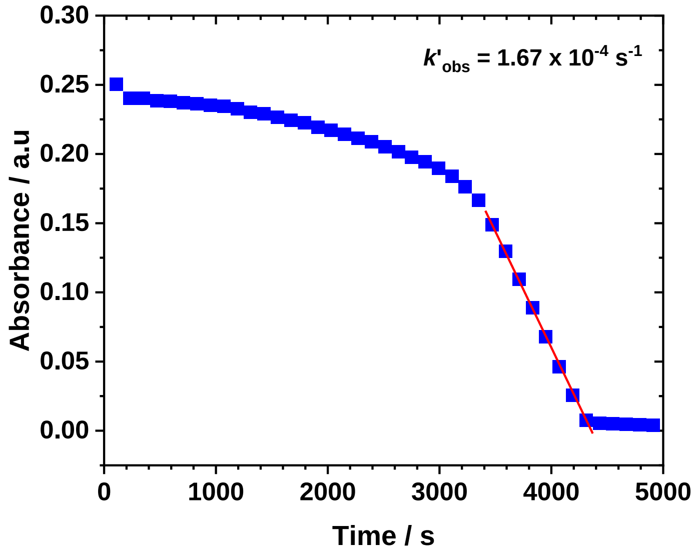

Figure S33: Plot of absorbance (1785 cm^{-1}) versus time for the ROCOP of NA/CHO using **3**.

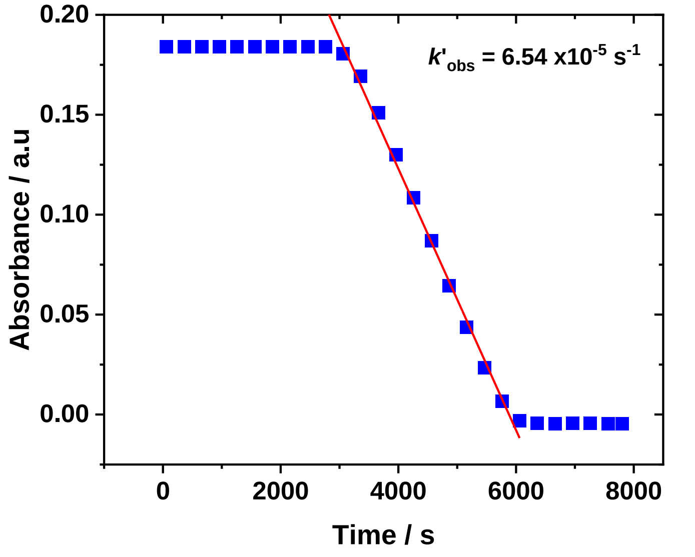

Figure S34: Plot of absorbance (1785 cm^{-1}) versus time for the ROCOP of NA/CHO using **4**.

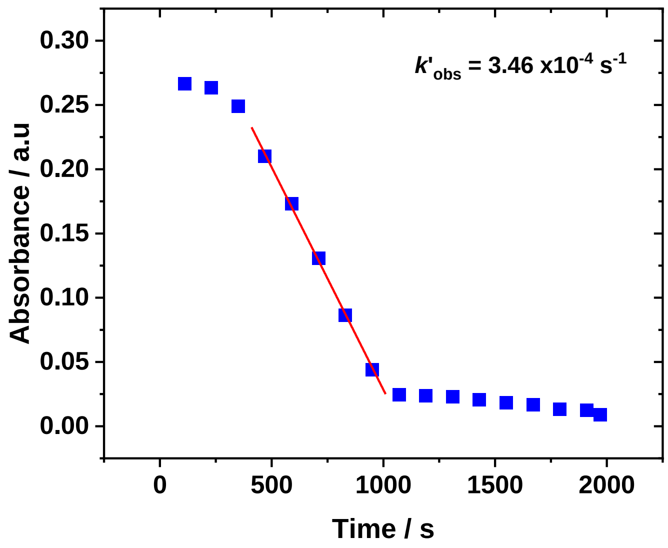

Figure S35: Plot of absorbance (1785 cm^{-1}) versus time for the ROCOP of NA/CHO using **5**.

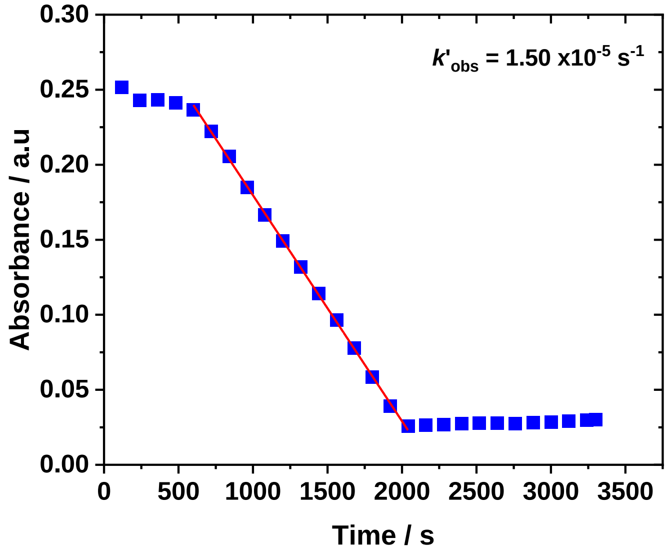

Figure S36: Plot of absorbance (1785 cm^{-1}) versus time for the ROCOP of NA/CHO using **6**.

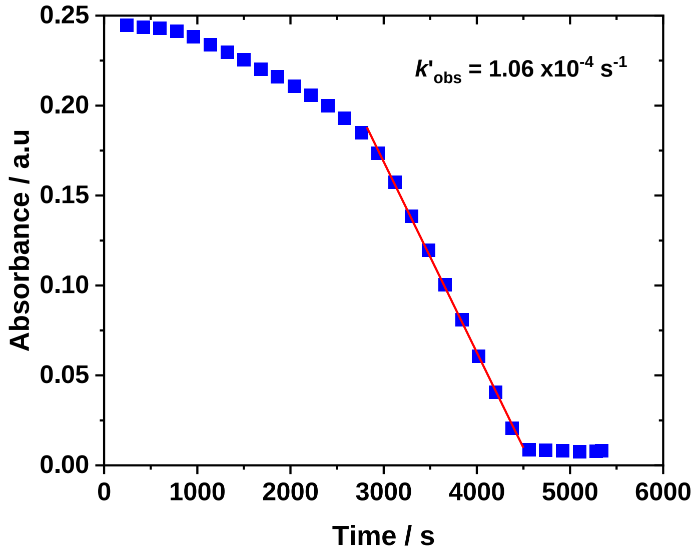

Figure S37: Plot of absorbance (1785 cm^{-1}) versus time for the ROCOP of NA/CHO using **7**.

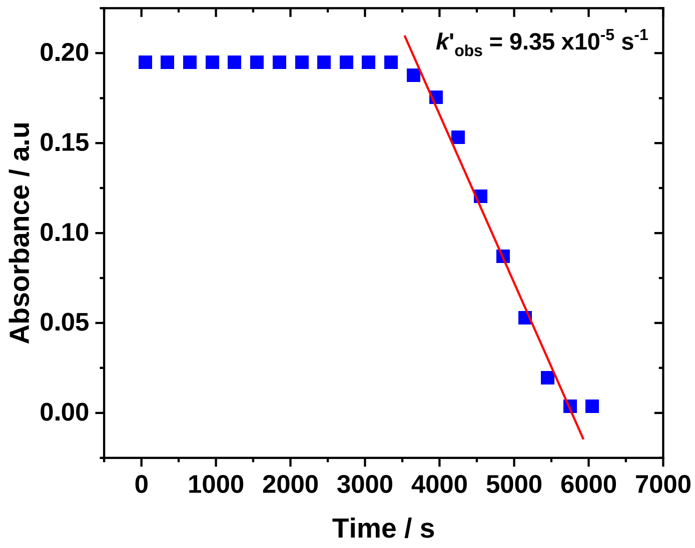

Figure S38: Plot of absorbance (1785 cm^{-1}) versus time for the ROCOP of NA/CHO using **8**.

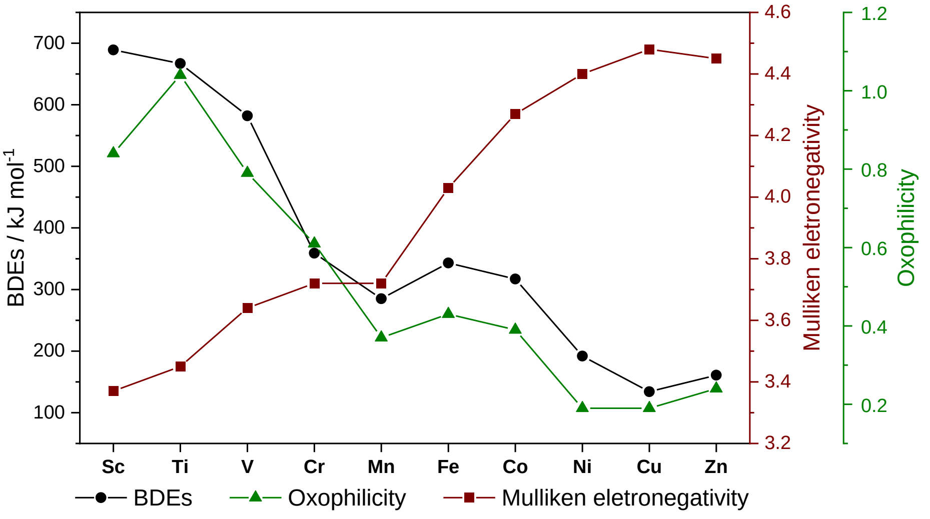

Figure S39: Plot of first row transition metals bond dissociation energies (BDEs), electronegativities (Mulliken) and oxophilicity.

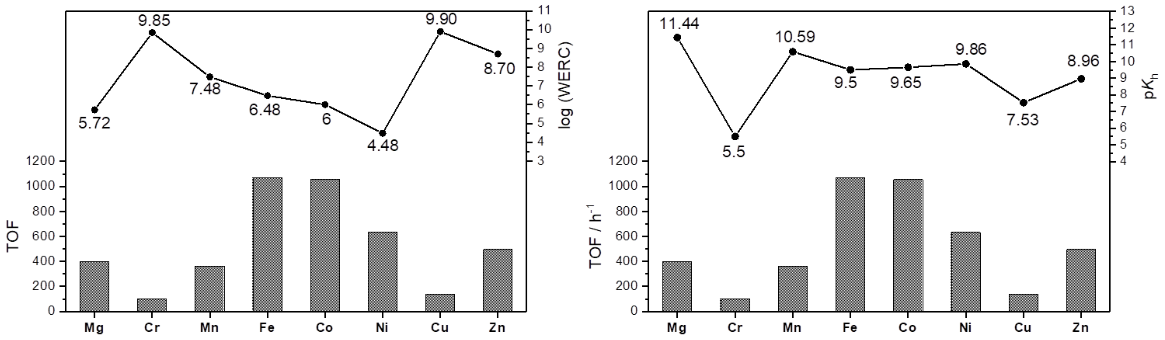

Figure S40: Plots of water exchange rate constants (LHS) and hydrolysis constants (RHS) against metal ion and their observed activity in copolymerisation of CO2/CHO.

Table S2: Experimental details for the crystallographic analysis of **Fe(II)Fe(III)**.

	SHELX
Crystal Data	
Chemical Formula	$C_{48}H_{79}Fe_2N_4O_{10}$
M_n	983.85
Crystal System, Space Group	Monoclinic, C2/c
Temperature (K)	150
a, *b*, *c* (Å)	16.5262 (2), 11.7955 (1), 26.4529 (4)
β (°)	104.599 (1)
V (Å^3)	4990.10
Z	4
Radiation Type	Cu κ_α
μ (mm^{-1})	5.14
Crystal Size (mm)	0.25 x 0.16 x 0.04
Data collection	
Diffractometer	SuperNova, Dual, Cu at zero, Atlas
Absorption Correction	Multi-scan CrysAlisPRO 1.171.38.43b (Rigaku Oxford Difrraction, 2015) Empirical absorption correction using spherical harmonics, implemented in SCALE3 ABSPACK scaling algorithm
T_{min}, T_{max}	0.652, 1
No. of measured, independent and observed [I > 2σ(I)] reflections	29910, 5209, 4635
R_{int}	0.051
(sin θ/λ)$_{max}$ (Å^{-1})	0.630
Refinement	
$R[F^2 > 2s(F^2)]$, $wR(F^2)$, S	0.038, 0.103, 1.03
No. of reflections	5209
No. of parameters	305
H-atom treatment	H atoms treated by a mixture of independent and constrained refinement
$\Delta\rho_{max}$, $\Delta\rho_{min}$ (e Å^{-3})	0.82, -0.33

References

1. Yuntawattana, N.; McGuire, T. M.; Durr, C. B.; Buchard, A.; Williams, C. K. *Catal. Sci. Tech*. **2020**, 10 7226

2. Thevenon, A.; Cyriac, A.; Myers, D.; White, A. J. P.; Durr, C. B.; Williams, C. K. *J. Am. Chem. Soc.* **2018**, 140, 6893.

3. Oxford Diffraction. CrysAlisPRO; Agilent Technologies UK Ltd: Yarnton, England.

4. Sheldrick, G. M. *Acta Crystallogr. Sect. Found. Adv*. **2015**, 71, 3

5. Sheldrick, G. M. *Acta Crystallogr. A.* **2008**, 64, 112

6. Farrugia, L. J. *J. Appl. Crystallogr*. **2012**, 45, 849

7. IUCR, CIF publishing tools https://publcif.iucr.org/publcif.php (accessed 2021 -09 -03)
